# Mutation‐Immune Crosstalk Contextualizes CAF‐Associated ARPC1A Programs and Cisplatin Response in Gastric Cancer

**DOI:** 10.1155/humu/9607360

**Published:** 2026-08-19

**Authors:** Deyi Lu, Fanlai Meng, Weiying Zhou, Yifei Hu, Gaosha Li, Jiahao Ge, Qing Zhang, Qi Min

**Affiliations:** ^1^ Department of Gastroenterology, Affiliated Jinhua Hospital, Zhejiang University School of Medicine, Jinhua, Zhejiang, China, zju.edu.cn; ^2^ Department of Pathology, Nanjing Drum Tower Hospital Group Suqian Hospital, Suqian, Jiangsu, China; ^3^ Department of Clinical Laboratory, Zhejiang Jinhua Guangfu Cancer Hospital, Jinhua, Zhejiang, China; ^4^ Department of Clinical Laboratory, Affiliated Jinhua Hospital, Zhejiang University School of Medicine, Jinhua, Zhejiang, China, zju.edu.cn; ^5^ Department of Hepatobiliary and Pancreatic Surgery, Affiliated Jinhua Hospital, Zhejiang University School of Medicine, Jinhua, Zhejiang, China, zju.edu.cn; ^6^ Department of Hepatology, Huai′an Fourth People′s Hospital, Huai′an, China; ^7^ Department of Oncology, The Second People′s Hospital of Huai′an, The Affiliated Huai′an Hospital of Xuzhou Medical University, Huai′an, China; ^8^ Nanjing University of Chinese Medicine, Nanjing, China, njucm.edu.cn

**Keywords:** ARPC1A, cancer-associated fibroblasts, cisplatin resistance, copy-number alteration, DepMap CRISPR screening, gastric cancer, single-cell RNA sequencing, tumor microenvironment

## Abstract

Cisplatin response in gastric cancer is shaped by malignant cell programs, stromal states, and mutation‐immune crosstalk, but the CAF subtypes linked to platinum tolerance and their epithelial effectors remain incompletely defined. We integrated single‐cell RNA sequencing, epithelial CNV‐like inference, fibroblast reclustering, CellChat analysis, signature scoring, hdWGCNA, CAF‐epithelial coupling, exploratory GSE14209 evaluation, TCGA‐STAD mutation/copy‐number contextualization, and DepMap CRISPR virtual knockout analysis, followed by experimental validation in AGS and HGC‐27 cells. IGF1+ CXCL12+ CAFs showed resistance‐supportive stromal features, and CAF‐epithelial coupling prioritized ARPC1A as a tumor epithelial candidate associated with this stromal program. In TCGA‐STAD, ARPC1A mutations were rare and consisted of three missense and two frameshift variants without a recurrent hotspot; the mutant tumors were confined to the MSI subtype and showed an immune‐activated, high TMB/MSI context, indicating mutation‐immune crosstalk rather than a clear mutation‐specific CAF, EMT, drug resistance, or cisplatin response phenotype. By contrast, ARPC1A copy‐number gain/amplification was more frequent and tracked with ARPC1A expression, CIN enrichment, aneuploidy, and fraction of genome altered. DepMap CRISPR data indicated mostly weak‐to‐mild baseline ARPC1A dependency in upper GI and gastric/GEJ models. Experimentally, ARPC1A knockdown increased cisplatin sensitivity, promoted cisplatin‐induced apoptosis, and suppressed migration and invasion. These findings identify a CAF‐associated ARPC1A epithelial program linked to cisplatin response and support a mutation‐immune/copy‐number framework in which ARPC1A is interpreted mainly through immune‐contextual mutation patterns and expression dosage rather than recurrent gain‐of‐function mutation.

## 1. Introduction

Gastric cancer remains a major cause of cancer‐related death worldwide [1], and systemic chemotherapy continues to be a cornerstone of treatment for advanced disease [2]. Cisplatin‐based therapy remains clinically important, but its efficacy is frequently limited by intrinsic or acquired resistance [3, 4]. In gastric cancer, therapeutic failure cannot be fully explained by tumor cell–intrinsic alterations alone. Increasing evidence indicates that treatment sensitivity is shaped within the tumor ecosystem, where malignant epithelial cells interact with stromal and immune compartments to support survival, adaptation, and phenotypic plasticity under drug pressure [5].

Among the nonmalignant components of this ecosystem, cancer‐associated fibroblasts have emerged as important regulators of therapeutic response [6]. Beyond their structural role, CAFs remodel the extracellular matrix, influence cytokine and growth factor signaling, and modulate inflammatory and immune states within the tumor microenvironment [7, 8]. These functions place CAFs in a position to promote drug tolerance and persistence of malignant cells [9]. However, CAF populations are highly heterogeneous, and their contributions to chemotherapy resistance are unlikely to be uniform across all fibroblast states [10]. A key unresolved issue in gastric cancer is therefore not simply whether CAFs participate in resistance, but which CAF subpopulations are most closely linked to resistance‐supportive programs and how they communicate with tumor epithelial cells during treatment adaptation [11].

Single‐cell transcriptomic approaches provide a useful framework for addressing this problem because they enable simultaneous dissection of malignant epithelial states, stromal heterogeneity, immune remodeling, and intercellular communication at cellular resolution [12]. This is particularly relevant in gastric cancer, where epithelial diversity and microenvironmental complexity are often obscured in bulk‐level analyses [13]. By integrating single‐cell analysis with epithelial CNV‐like inference, fibroblast reclustering, cell–cell communication modeling, coexpression network analysis, and cautious external treatment‐cohort evaluation, it becomes possible to move beyond descriptive profiling and prioritize stromal states associated with pharmacologic resistance [14, 15].

In parallel, the copy‐number and broader genomic alteration landscapes of gastric cancer provide an important context for interpreting microenvironmental coupling. Somatic alterations can shape tumor immunogenicity, molecular subtype, chromosomal instability, and therapeutic vulnerability, whereas low‐frequency point mutations in an individual candidate gene may coexist with broader dosage alterations such as copy‐number gain or amplification. Integrating microenvironmental analysis with genomic context can therefore clarify whether a CAF‐linked epithelial candidate is primarily defined by recurrent point mutation, copy‐number dosage, molecular subtype, or transcriptional coupling [16].

In the present study, we used a tumor‐ecosystem pharmacology framework to examine how stromal programs are associated with cisplatin response biology in gastric cancer. We identified an IGF1+ CXCL12+ CAF population enriched for resistance‐supportive programs, mapped its communication with epithelial tumor cells, and associated this stromal program with ARPC1A as a candidate epithelial effector. We further placed ARPC1A within the TCGA‐STAD copy‐number and genomic alteration context and used DepMap CRISPR screening data as a virtual knockout resource before testing whether ARPC1A contributes to cisplatin sensitivity and malignant phenotypes in two gastric cancer cell lines [17]. Together, these analyses connect single‐cell microenvironmental states, genomic context, functional dependency information, and experimentally measurable drug‐response behavior.

## 2. Methods

### 2.1. Data Collection and Preprocessing

Single‐cell RNA‐sequencing data were obtained from the Gene Expression Omnibus (GEO) under Accession Number GSE206785 [18]. This dataset contains paired tumor and adjacent normal gastric tissue samples from 24 patients, yielding 48 samples in total. The processed expression matrix provided by the original study was used for downstream analyses. Because the public dataset had already undergone SCTransform‐based normalization, subsequent analyses were performed on the processed expression matrix after recalculation of quality control metrics and downstream re‐embedding [19]. After quality control, the final dataset included 105,299 cells across all samples. For external treatment‐cohort evaluation, we used the paired bulk transcriptome cohort GSE14209, which includes gastric cancer biopsies collected before and after cisplatin and fluorouracil (CF) combination chemotherapy [20]. This dataset belongs to the GEO SuperSeries GSE14210, in which GSE14208 captures clinical response to CF chemotherapy and GSE14209 focuses on chemotherapy‐related transcriptional changes [21]. Probe‐level expression values were collapsed to gene‐level values according to platform annotation, and only patients with matched pretreatment and posttreatment samples were retained for paired analyses.

### 2.2. Quality Control, Dimensionality Reduction, and Cell Type Annotation

All single‐cell analyses were performed in R (V4.2.0) using the Seurat package (V4.0) [22]. For each cell, the number of detected genes, total expression counts, and mitochondrial transcript proportion were recalculated. Cells were retained if they satisfied the following criteria: 400 ≤ nFeature ≤ 3000 and percent.mt ≤ 10%. After filtering, 3000 highly variable genes were identified, followed by data scaling and principal component analysis (PCA). To reduce intersample batch effects, Harmony integration was performed using sample identity as the grouping variable [23]. Neighborhood graph construction, clustering, and UMAP visualization were then carried out on the first 30 Harmony dimensions, with clustering performed at resolution = 0.2. Cell type annotation was based on the original dataset annotations and further checked using canonical marker genes through FeaturePlot, DotPlot, and heatmap visualization. This procedure generated a global atlas containing 12 major cell lineages, including epithelial cells, fibroblasts, endothelial cells, B cells, CD4+ T cells, CD8+ T cells, myeloid cells, plasma cells, mural cells, mast cells, innate lymphoid cells, and glial cells.

### 2.3. Global Tumor Microenvironment Analysis

To characterize tumor microenvironment remodeling, cell type proportions were summarized at both the sample and patient levels. For patients with paired tumor and normal tissues, differences in lineage composition were evaluated using paired Wilcoxon signed‐rank tests, followed by Benjamini–Hochberg (BH) multiple‐testing correction. For major cell lineages, pseudobulk expression matrices were generated by averaging expression values within each sample. Tumor‐versus‐normal differential expression analyses were then performed using limma [24], requiring each lineage to contain at least 100 cells, with at least 30 cells per group and at least two eligible samples per condition. Ranked gene statistics were used for fgsea‐based enrichment analysis against Hallmark and Gene Ontology Biological Process gene sets. In parallel, predefined hallmark and custom functional gene sets were used to calculate representative program scores across the atlas, including proliferation, cell cycle, epithelial–mesenchymal transition (EMT), hypoxia, angiogenesis, inflammatory response, CAF activation, ECM remodeling, antigen presentation, cytotoxicity, and T‐cell exhaustion.

### 2.4. Epithelial Cell Reclustering, CNV‐Like Inference, and Malignant Cell Identification

Epithelial cells were extracted from the global atlas and reanalyzed separately to obtain a refined epithelial state landscape. Based on the existing integrated embedding, an epithelial‐specific neighbor graph and UMAP were reconstructed for higher resolution analysis. To infer malignant epithelial cells, we established a reference‐centered CNV‐like scoring framework using normal epithelial cells as the reference population. This approach was used as an expression‐based proxy for large‐scale chromosomal deviation rather than as direct genomic CNV measurement. Genomic coordinates of autosomal genes were obtained from org.Hs.eg.db. Genes detected in at least 5% of both all epithelial cells and reference normal epithelial cells were retained. Genes were then ordered by chromosomal position and grouped into bins of 100 genes per bin. For each cell, average expression within each bin was calculated, centered to the mean of reference normal epithelial cells, standardized by the corresponding standard deviation (SD), and smoothed along chromosomes using a sliding window of Size 3 to generate CNV‐like signals. Cell‐level CNV‐like burden was defined as the mean absolute smoothed bin signal. Candidate malignant cells were identified using empirical thresholds derived from the normal epithelial reference distribution: the 95th percentile as the candidate threshold and the larger value between the 99th percentile and median + 3 × MAD as the high‐confidence threshold. To further constrain high CNV‐like epithelial cells, a two‐center *k*‐means procedure was applied within tumor‐derived epithelial cells. Epithelial cells were ultimately classified as reference normal, tumor CNV‐like‐low, tumor candidate, or tumor high‐confidence groups.

### 2.5. Characterization of Tumor Epithelial States

After malignant epithelial cell identification, tumor‐related epithelial programs were characterized at both the cell and sample levels. First, tumor cells were compared with nontumor epithelial cells at the single‐cell level. Second, within tumor samples, sample‐level averaged expression profiles were generated to compare tumor cells with tumor CNV‐low epithelial cells in a paired pseudobulk framework, thereby reducing confounding from intersample compositional heterogeneity. Differential expression results from these two levels were integrated to prioritize directionally consistent markers. To define functional states of malignant epithelial cells, we quantified multiple tumor‐associated programs, including proliferation, cell cycle, EMT, hypoxia, DNA repair, p53 pathway activity, MYC targets, TGF‐beta signaling, angiogenesis, a custom gastric tumor program, and stemness‐related programs. These signatures were used to depict the functional landscape of epithelial malignancy.

### 2.6. Fibroblast Reclustering and CAF Subtype Annotation

Fibroblasts were first examined within the original global annotation framework to assess their tissue distribution, tumor‐versus‐normal transcriptional differences, enriched biological programs, and marker landscape. Fibroblasts were then extracted for dedicated reclustering analysis. In this fibroblast‐focused analysis, 1000 highly variable genes were selected, followed by scaling, PCA, Harmony integration, UMAP embedding, and neighbor graph construction based on the first 20 Harmony dimensions. Clustering was performed at resolution = 0.3. Clusters were manually annotated according to top marker genes and known fibroblast/CAF features, resulting in five major fibroblast/CAF subtypes: resident fibroblasts, IGF1+ CXCL12+ CAFs, HAS1+ inflammatory CAFs, POSTN+ matrix CAFs, and ASPN+ desmoplastic CAFs. The IGF1+ CXCL12+ CAF state shared selected inflammatory and secretory features with previously described iCAF‐like programs, but it was not treated as identical to canonical iCAF or myCAF categories because it also carried CXCL12‐retention, IGF1‐axis, and resistance‐supportive signatures in this dataset. Tumor‐versus‐normal composition changes, paired patient‐level abundance shifts, subtype‐versus‐rest differential expression, and selected functional program enrichments were used to establish the biological heterogeneity of CAF states and their relationship to resistance‐associated features.

### 2.7. Functional Enrichment and Signature Scoring

For fibroblast/CAF analyses, detailed characterization of IGF1+ CXCL12+ CAFs, and cross‐compartment comparisons, signature scores were calculated as the average expression of genes in each predefined gene set at the single‐cell or sample average level. CAF‐related functional programs included EMT, TGF‐beta signaling, ECM/collagen remodeling, angiogenesis, hypoxia, inflammatory response, TNF‐alpha/NF‐*κ*B signaling, wound healing, focal adhesion/integrin signaling, drug barrier ECM, glycolysis, and survival signaling. The component signatures were selected from established CAF biology, chemotherapy‐resistance literature, and programs observed in the subtype‐level enrichment analysis. To assess resistance‐related stromal features, several known or candidate CAF‐associated signatures were additionally incorporated, including IGF1_CAF_signature, PPIC_labeled_CAF_signature, and C3_complement_CAF_signature, as well as IGF1+ CAF‐focused signatures such as IGF1_axis, CXCL12_retention, partial_EMT_support, stress_adaptation, stemness_niche, and complement_stroma. Each component score was standardized, and the standardized values were averaged to generate the composite resistance_support_index. This index was used as a summary measure of resistance‐supporting stromal tendencies rather than as a direct measurement of clinical drug resistance.

### 2.8. Cell–Cell Communication Analysis

To investigate stromal–epithelial signaling, CellChat analysis was performed using major microenvironmental compartments, including tumor epithelial, nontumor epithelial, normal epithelial, fibroblast, endothelial, mural, myeloid, T‐cell, B‐cell, plasma, innate lymphoid, and mast cell groups [25]. To avoid dominance by large cell populations, a sample‐stratified quota downsampling strategy was applied to balance input cell numbers while preserving the relative sample composition as much as possible. Cell–cell communication probabilities were then inferred using CellChat with the human interaction database, trimean‐based communication probability estimation, and bootstrap testing to identify significant ligand–receptor pairs, pathway activity, network strength, and centrality. Communication analysis was further extended to the CAF subtype level. In this subtype‐aware analysis, the five CAF subtypes were analyzed together with tumor epithelial, nontumor epithelial, T/NK, myeloid, endothelial, and other major target populations after an additional round of stratified downsampling. Particular attention was paid to resistance‐relevant signaling axes, including IGF, CXCL, CCL, IL‐6, collagen, laminin, FN1, TGF‐*β*, and THBS pathways.

### 2.9. Deep Characterization of IGF1+ CXCL12+ CAFs and hdWGCNA Analysis

IGF1+ CXCL12+ CAFs were directly compared with all other fibroblasts, and tumor‐versus‐normal comparisons were further performed within this subtype. Marker analysis, GSEA, paired abundance changes, and multiprogram heatmaps were used to define the molecular identity and intersample heterogeneity of this CAF state. To identify internal coexpression modules associated with resistance‐supportive behavior, hdWGCNA was performed within IGF1+ CXCL12+ CAFs [26, 27]. Briefly, 1200 highly variable genes were selected after removing mitochondrial and ribosomal genes. Soft‐thresholding powers from 1 to 8 were evaluated, and the first power satisfying the scale‐free topology criterion was selected. Coexpression modules were then constructed using minModuleSize = 30. Module eigengenes were correlated with resistance_support_index, EMT, hypoxia, glycolysis, drug barrier ECM, known CAF signatures, and tumor‐versus‐normal status using Spearman correlation. Module membership (kME), gene significance, and hub scores were integrated to prioritize key resistance‐related modules and hub genes. These module and hub‐gene signals were further projected onto the external bulk dataset as exploratory support for whether the single‐cell‐derived stromal programs were detectable in an independent chemotherapy‐related cohort.

### 2.10. CAF‐Epithelial Coupling Analysis

To assess cross‐compartment resistance coupling, a sample‐level CAF resistance axis was constructed by integrating the key hdWGCNA module signal, hub‐gene projection scores, IGF1+ CAF abundance, and known IGF1‐related CAF signatures. On the epithelial side, tumor epithelial cells were scored for EMT, hypoxia, DNA repair, stemness, gastric tumor program, partial EMT, and survival signaling, which were further summarized as an epithelial resistance index. Within shared tumor samples, Spearman correlation was used to quantify the relationships between CAF‐side indicators and epithelial programs. Tumor epithelial pseudobulk matrices were then generated at the sample level, and each epithelial gene was correlated with both the CAF resistance axis and the epithelial resistance index. To prioritize genes consistent with malignant epithelial states, these sample‐level correlations were integrated with tumor cell versus nontumor epithelial differential expression results, thereby defining high‐confidence, supportive, and rank‐based candidate genes. ARPC1A was selected for downstream validation because it combined positive CAF‐axis coupling, epithelial resistance index association, preferential tumor epithelial expression, supportive TCGA expression and survival patterns, and experimental feasibility for loss‐of‐function assays. Candidate CAF‐coupled epithelial genes were subsequently subjected to fgsea analysis and visualized using heatmaps, FeaturePlots, and DotPlots.

### 2.11. External Bulk Transcriptome Validation

To evaluate whether stromal programs identified in the single‐cell data could also be detected in an independent chemotherapy‐related setting, we analyzed GSE14209 as an exploratory external paired bulk cohort associated with CF chemotherapy in gastric cancer. The dataset contains matched biopsy samples collected before and after CF‐based chemotherapy. In the bulk cohort, we calculated sample‐level scores for IGF1_CXCL12_CAF_core, IGF1_CXCL12_CAF_extended, C3_complement_CAF, ECM_barrier, drug_resistance, and the integrated CAF_resistance_combined signature. Differential analysis was performed using a paired limma design, with patient identity included as a blocking factor and treatment phase (pretreatment vs. posttreatment) as the variable of interest. Ranked gene statistics were further used for fgsea analysis against Hallmark and custom CAF/resistance‐related pathways. For selected marker genes, paired Wilcoxon signed‐rank tests were additionally performed. Because this cohort is bulk level, modest in size, and not designed to isolate CAF subtypes directly, these analyses were interpreted as supportive pathway‐level evidence rather than definitive validation of a single CAF population. The paired design is consistent with prior use of this dataset as a pre/postchemotherapy gastric cancer cohort with 22 patients analyzed twice.

### 2.12. TCGA‐STAD Genomic Alteration and DepMap CRISPR Virtual Knockout Analysis

TCGA‐STAD Pan‐Cancer Atlas data were accessed through the cBioPortal API to contextualize ARPC1A within the gastric cancer copy‐number and genomic alteration landscape [28, 29]. Somatic mutation data were retrieved for ARPC1A and representative gastric cancer driver or context genes, and mutation frequencies were calculated using the sequenced sample set. ARPC1A variants were classified by sequence consequence as missense or truncating frameshift events, mapped to protein position, and reviewed for recurrence, hotspot formation, and coexisting copy‐number/expression context. Because these data do not include allele‐specific functional assays, missense mutations were treated as variants of uncertain functional consequence, whereas frameshift variants were considered compatible with loss‐of‐function biology. ARPC1A copy‐number status was obtained from GISTIC calls and categorized as loss, diploid, gain, or amplification. To evaluate mutation‐phenotype relationships, samples were assigned to mutually exclusive ARPC1A genomic states: mutant, amplification, gain, or neutral/loss. ARPC1A mRNA *z*‐scores, molecular subtype annotations, tumor mutational burden, MSIsensor score, aneuploidy score, fraction genome altered, CAF‐related signatures, immune signatures, EMT‐like score, and drug‐resistance score were integrated at the sample level when available. Differences in continuous variables across alteration groups were assessed using nonparametric tests, and associations between ARPC1A copy‐number status and mRNA expression were evaluated using Spearman correlation. These bulk TCGA data cannot identify ARPC1A‐mutated CAF subpopulations directly; therefore, mutation‐associated CAF and immune effects were assessed using tumor‐level CAF and immune signature scores as indirect microenvironmental readouts. To approximate the baseline fitness consequence of ARPC1A depletion in cancer models, DepMap Public 24Q2 CRISPRGeneEffect and Model metadata were analyzed as a virtual knockout resource. The ARPC1A (10552) CRISPR gene‐effect score was extracted for all screened cell line models and merged with model lineage and disease annotations. Upper GI models were defined from DepMap lineage and disease metadata, and gastric/GEJ models were treated as a strict subset. Gene‐effect distributions were summarized across all screened models, upper GI models, and gastric/GEJ models using standard DepMap dependency thresholds (mild dependency, gene effect < −0.2; moderate dependency, gene effect < −0.5; strong dependency, gene effect < −1.0). Study‐relevant gastric cancer cell lines, including AGS, HGC‐27, MKN‐45, NCI‐N87, SNU‐16, and KATO III, were additionally highlighted.

### 2.13. Cell Culture

Human gastric cancer cell lines AGS (RRID:CVCL_0139) and HGC‐27 (RRID:CVCL_1279), the normal gastric epithelial cell line GES‐1 (RRID:CVCL_0H23), and human embryonic kidney cell HEK293T were obtained from the Cell Bank of the Chinese Academy of Sciences (Shanghai, China). AGS and HGC‐27 cells were maintained in RPMI‐1640 medium (Gibco, Cat# 11875‐093), whereas GES‐1 and HEK293T cells were cultured in Dulbecco′s Modified Eagle Medium (DMEM, Gibco, Cat# 11965‐092), both supplemented with 10% fetal bovine serum (FBS; Gibco, Cat# 10099‐141) and 1% penicillin–streptomycin solution (Gibco, Cat# 15140‐122). All cells were incubated at 37°C in a humidified atmosphere containing 5% CO_2_ and were routinely passaged at 70%–80% confluence. Cells used in experiments were confirmed to be free of mycoplasma contamination. Cell line identities were based on authenticated cell bank stocks; no additional STR reauthentication was performed in our laboratory immediately before these experiments.

### 2.14. Stable Cell Line Construction and Validation

Stable ARPC1A knockdown cell lines were generated using short hairpin RNA (shRNA) lentiviral transduction. Briefly, shRNA sequences targeting ARPC1A and a nontargeting control (shNC) were cloned into a lentiviral vector (GeneChem, Shanghai, China). Lentiviral particles were produced by cotransfecting HEK293T cells with the shRNA plasmid and packaging plasmids using Lipofectamine 3000 (Thermo Fisher Scientific, Cat# L3000015). Viral supernatants were collected, filtered, and used to infect AGS and HGC‐27 cells in the presence of Polybrene (Sigma‐Aldrich, Cat# TR‐1003‐G). After 48 h, infected cells were selected with puromycin (Sigma‐Aldrich, Cat# P8833) to establish stable cell lines. The knockdown efficiency of ARPC1A was evaluated by quantitative real‐time PCR (qRT‐PCR), and clones with the highest knockdown efficiency were selected for subsequent functional assays.

### 2.15. qRT‐PCR

Total RNA was isolated from cells using TRIzol Reagent (Thermo Fisher Scientific, Cat# 15596026) according to the manufacturer′s instructions. RNA concentration and purity were assessed by spectrophotometry. Reverse transcription was performed using the PrimeScript RT Reagent Kit with gDNA Eraser (Takara, Cat# RR047A) to eliminate genomic DNA contamination and synthesize cDNA. qRT‐PCR was carried out using TB Green Premix Ex Taq II (Takara, Cat# RR820A) on a QuantStudio 5 Real‐Time PCR System (Applied Biosystems). Amplification conditions followed the manufacturer′s protocol, and each sample was analyzed in triplicate. GAPDH was used as the internal reference gene for normalization. Relative gene expression levels were calculated using the 2^−*ΔΔ*Ct^ method. Primer sequences used in this study were as follows:

GAPDH‐F: TTCTTTTGCGTCGCCAGCC

GAPDH‐R: TCCCGTTCTCAGCCTTGAC

ARPC1A‐F: CCAGCATGACAAACTGTGGC

ARPC1A‐R: CGGCATTGGCGTGATATGTG

ABCB1‐F: CGGCTCTTCCAAGCTCAAAG

ABCB1‐R: CATTGCGGTCCCCTTCAAGA

ABCC1‐F: GGACTTCGTTCTCAGGCACA

ABCC1‐R: TCCTGGGGGATGATGGTGAT

BCL2‐F: AAAAATACAACATCACAGAGGAAGT

BCL2‐R: GTTTCCCCCTTGGCATGAGA

MCL1‐F: CACTTCCGCTTCCTTCCAGT

MCL1‐R: CCTCCTTCTCCGTAGCCAAA

CDH1‐F: AGTGACTGATGCTGATGCCC

CDH1‐R: AATGTACTGCTGCTTGGCCT

VIM‐F: TCACCTGTGAAGTGGATGCC

VIM‐R: ACGAAGGTGACGAGCCATTT

### 2.16. Cell Viability Assay (Cell Counting Kit‐8 [CCK‐8])

Cell viability was evaluated using a CCK‐8 (Dojindo, Cat# CK04) according to the manufacturer′s protocol. Briefly, AGS and HGC‐27 cells with stable ARPC1A knockdown or control cells were seeded into 96‐well plates at a density of 3–5 × 10^3^ cells per well and allowed to adhere overnight. Cells were then treated with a series of cisplatin (CDDP; Sigma‐Aldrich, Cat# P4394) concentrations for 24–48 h. After treatment, 10 *μ*L of CCK‐8 solution was added to each well and incubated at 37°C for 1–2 h. The absorbance was measured at 450 nm using a microplate reader (BioTek, United States). Cell viability was calculated as a percentage relative to untreated control cells. Dose–response curves were generated, and the half‐maximal inhibitory concentration (IC50) values were calculated using GraphPad Prism software (Version 9.0).

### 2.17. Cell Colony Formation

For the colony formation assay, AGS and HGC‐27 cells with stable ARPC1A knockdown or control cells were seeded into 6‐well plates at a density of 1000 cells per well and cultured under standard conditions. After cell attachment, cells were treated with cisplatin (CDDP; Sigma‐Aldrich, Cat# P4394) at a final concentration of 3 *μ*M or left untreated. The culture medium was replaced every 2–3 days, and cells were maintained for 10–14 days to allow colony formation. Colonies were then fixed with 4% paraformaldehyde (Biosharp, Cat# BL539A) for 15 min and stained with 0.1% crystal violet solution (Solarbio, Cat# G1062) for 20 min at room temperature. After washing with phosphate‐buffered saline (PBS; Gibco, Cat# 10010‐023), plates were air‐dried, and colonies consisting of more than 50 cells were counted under a microscope. Each experiment was performed in triplicate.

### 2.18. Apoptosis Analysis by Flow Cytometry

Cell apoptosis was evaluated using an Annexin V‐FITC/PI apoptosis detection kit (BD Biosciences, Cat# 556547) following the manufacturer′s instructions. Briefly, AGS and HGC‐27 cells with stable ARPC1A knockdown or control cells were seeded into 6‐well plates and treated with cisplatin (CDDP; Sigma‐Aldrich, Cat# P4394) at a final concentration of 5 *μ*M for 24 h, whereas untreated cells served as controls. After treatment, cells were harvested, washed twice with cold PBS (Gibco, Cat# 10010‐023), and resuspended in 1× binding buffer. Subsequently, cells were incubated with Annexin V‐FITC and propidium iodide (PI) at room temperature for 15 min in the dark. Stained cells were immediately analyzed using a flow cytometer (BD FACSCanto II, BD Biosciences). Early and late apoptotic cells were combined to calculate the total apoptosis rate. Each experiment was performed independently at least three times.

### 2.19. Wound‐Healing Assay

Cell migration was evaluated using a wound‐healing assay. Briefly, AGS and HGC‐27 cells with stable ARPC1A knockdown or control cells were seeded into 6‐well plates and cultured to near 90%–100% confluence. Cells were pretreated with Mitomycin C (10 *μ*g/mL; Sigma‐Aldrich) for 2 h to inhibit proliferation. A linear wound was generated using a sterile 200 *μ*L pipette tip, and detached cells were gently removed by washing with PBS (Gibco, Cat# 10010‐023). To minimize the influence of cell proliferation, cells were maintained in serum‐free medium or medium containing low serum (1% FBS; Gibco, Cat# 10099‐141). Images of the same fields were captured at 0 and 24 h using an inverted microscope (Olympus, Japan). The wound area was measured using ImageJ software, and the migration rate was calculated as follows: (initial wound area − wound area at 24 h)/ initial wound area × 100%. Each experiment was independently repeated at least three times.

### 2.20. Transwell Invasion Assay

Cell invasion ability was assessed using Transwell chambers with Matrigel coating (Corning, 8 *μ*m pore size, Cat# 3422). Briefly, the upper chambers were precoated with Matrigel (BD Biosciences, Cat# 356234) diluted in serum‐free medium and allowed to solidify at 37°C. AGS and HGC‐27 cells with stable ARPC1A knockdown or control cells were resuspended in serum‐free medium, and 5 × 10^4^–1 × 10^5^ cells were seeded into the upper chamber. The lower chamber was filled with medium containing 10% FBS (Gibco, Cat# 10099‐141) as a chemoattractant. After incubation for 24–48 h at 37°C, noninvading cells on the upper surface were gently removed with a cotton swab, whereas cells that had invaded the lower surface of the membrane were fixed with 4% paraformaldehyde (Biosharp, Cat# BL539A) for 15 min and stained with 2% crystal violet (Solarbio, Cat# G1062) for 20 min. Images were captured under an inverted microscope (Olympus, Japan), and invaded cells were quantified using ImageJ software. Each experiment was performed in triplicate.

### 2.21. Statistical Analysis

All statistical analyses were performed using GraphPad Prism (Version 9.0; GraphPad Software, United States), R software (Version 4.2.0), and Python‐based statistical routines where appropriate. Quantitative experimental data are presented as mean ± SD from at least three independent experiments. For experimental assays, comparisons between two groups were conducted using an unpaired two‐tailed Student′s *t*‐test, whereas multiple‐group comparisons were analyzed by one‐way ANOVA followed by Tukey′s post hoc test. For bioinformatics and TCGA‐STAD genomic analyses, data distributions were visualized using boxplots, violin plots, heatmaps, lollipop plots, and alteration summary plots. Group comparisons were performed using the Wilcoxon rank‐sum test, Mann–Whitney *U* test, paired Wilcoxon signed‐rank test, or Kruskal–Wallis test, as appropriate. Correlation analyses were conducted using Spearman′s rank correlation coefficient. Multiple‐testing correction was performed using the BH method where applicable. A two‐sided *p* value < 0.05 was considered statistically significant. Significance levels were defined as  ^∗^ *p* < 0.05,  ^∗∗^ *p* < 0.01,  ^∗∗∗^ *p* < 0.001, and  ^∗∗∗∗^ *p* < 0.0001.

## 3. Results

### 3.1. Construction of a Global Single‐Cell Atlas of Gastric Cancer Tissues

**Figure 1:**
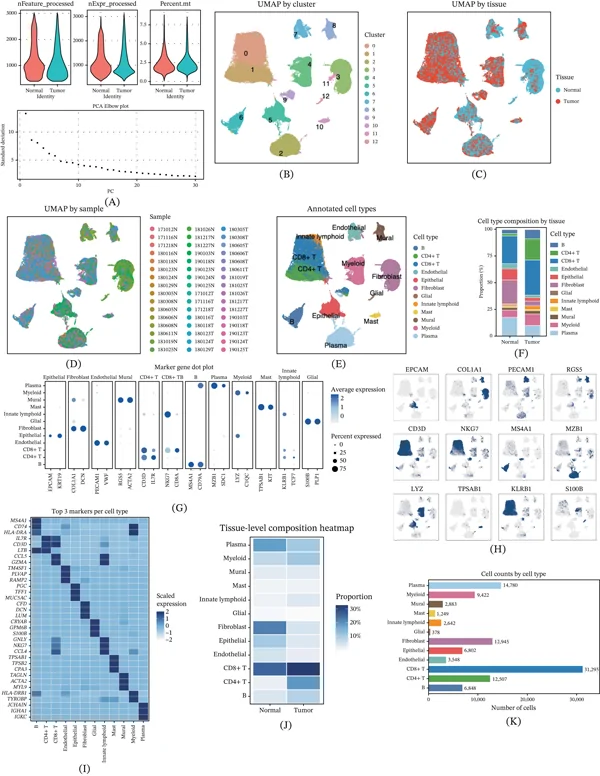
Construction of a global single‐cell atlas of gastric cancer tissues. (a) Quality control metrics of the integrated single‐cell dataset. Violin plots show the distributions of detected genes, total transcript counts, and mitochondrial transcript proportions in normal and tumor tissues. The elbow plot shows the principal components retained for downstream analysis. (b) UMAP visualization of the integrated dataset showing 13 transcriptionally distinct clusters. (c) UMAP colored by tissue origin, showing the distribution of cells from normal and tumor tissues. (d) UMAP colored by sample identity, showing the distribution of cells across individual samples after integration. (e) UMAP showing annotation of the major cell lineages, including B cells, CD4+ T cells, CD8+ T cells, endothelial cells, epithelial cells, fibroblasts, glial cells, innate lymphoid cells, mast cells, mural cells, myeloid cells, and plasma cells. (f) Relative proportions of major cell lineages in normal and tumor tissues. (g) Dot plot showing the expression of representative marker genes across annotated cell types. Dot size indicates the fraction of expressing cells, and color intensity indicates average expression level. (h) Feature plots showing the UMAP distribution of representative lineage markers. (i) Heatmap showing the top marker genes for each annotated cell lineage. (j) Heatmap showing the tissue‐level composition of major cell types in normal and tumor samples. (k) Absolute cell numbers of the major annotated cell lineages.

We first constructed a global single‐cell transcriptomic atlas from paired tumor and adjacent normal tissues in the GSE206785 cohort. After quality control, 105,299 cells from 48 samples of 24 patients were retained for downstream analysis. The overall distributions of detected genes, total transcript counts, and mitochondrial transcript proportions were acceptable in both normal and tumor tissues, supporting the quality of the dataset for subsequent integration and clustering. Principal component inspection indicated that the major transcriptional variation was captured within the leading dimensions used for downstream analysis (Figure 1A). Harmony‐based integration followed by unsupervised clustering identified 13 transcriptionally distinct cell clusters in the global atlas (Figure 1B). UMAP visualization showed that cells from tumor and normal tissues were partially intermixed but also redistributed across specific regions of the embedding, suggesting that tissue‐associated remodeling occurred within a conserved multicellular framework (Figure 1C). When colored by sample identity, cells from different samples were broadly distributed across shared transcriptional spaces rather than forming sample‐restricted aggregates, indicating that batch effects had been effectively controlled (Figure 1D). Based on canonical marker genes and cluster‐level expression patterns, the integrated atlas was annotated into 12 major cell lineages, including epithelial cells, fibroblasts, endothelial cells, mural cells, myeloid cells, B cells, CD4+ T cells, CD8+ T cells, plasma cells, mast cells, innate lymphoid cells, and glial cells (Figure 1E). The annotation was supported by lineage‐restricted marker expression in the dot plot and corresponding spatial distribution in the UMAP feature plots (Figure 1G,H). Comparison of tissue composition revealed clear differences between tumor and normal samples, with epithelial and fibroblast compartments relatively expanded in tumor tissues, whereas several immune populations were more represented in normal tissues (Figure 1F,J,K). The heatmap of representative lineage markers further supported the robustness of cell type annotation across the global atlas (Figure 1I). This atlas provided the framework for subsequent analyses of malignant epithelial states, stromal heterogeneity, and resistance‐associated interactions within the gastric cancer microenvironment.

### 3.2. Gastric Cancer Tissues Showed Coordinated Remodeling of Cellular Composition and Functional Programs

At the sample level, the cellular composition of gastric cancer tissues showed marked heterogeneity across patients (Supporting Information 1: Figure S1A). Nevertheless, paired comparison at the patient level revealed consistent shifts between tumor and adjacent normal tissues. Tumor samples showed relative expansion of epithelial and fibroblast compartments, whereas several immune populations, including CD8+ T cells, B cells, plasma cells, and endothelial cells, were reduced in tumors. By contrast, CD4+ T cells and myeloid cells showed a trend toward higher representation in tumor tissues (Supporting Information 1: Figure S1B). These findings indicate that gastric cancer progression is accompanied by coordinated changes in both stromal and immune compartments rather than epithelial expansion alone. Functional analysis showed clear lineage‐specific remodeling across the tumor ecosystem. Hallmark enrichment indicated that the epithelial compartment was preferentially associated with proliferative, metabolic, and EMT‐related programs, whereas CD4+ T cells and myeloid cells were enriched for inflammatory and cytokine‐responsive pathways (Supporting Information 1: Figure S1C,D,E,F,G). UMAP‐based signature mapping and sample‐level comparisons showed that EMT, hypoxia, CAF activation, and angiogenesis were mainly concentrated in epithelial and stromal compartments, whereas cytotoxicity and antigen presentation–related programs were largely restricted to immune cells (Supporting Information 1: Figure S1H,I,J). Within the epithelial compartment, tumor‐associated cells showed higher hypoxia, MYC targets, and proliferation‐related activity, whereas immune signatures displayed marked cell type specificity rather than uniform activation or suppression across all immune populations (Supporting Information 1: Figure S1K,L,M,N,O). The summary heatmaps further highlighted tumor‐associated increases in stromal activation, proliferative signaling, and selected resistance‐relevant programs (Supporting Information 1: Figure S1Q), supporting the presence of coordinated compositional and functional remodeling in gastric cancer tissues.

### 3.3. Reference‐Centered CNV‐Like Inference Identified Malignant Epithelial States in Gastric Cancer

To distinguish malignant epithelial cells from nonmalignant epithelial populations, we performed an epithelial‐focused reanalysis and established a reference‐centered CNV‐like inference framework using normal epithelial cells as the baseline. UMAP visualization showed that tumor‐ and normal‐derived epithelial cells were partially separated in the epithelial compartment, and the inferred CNV‐like score was clearly elevated in a subset of tumor‐derived cells (Supporting Information 2: Figure S2A,B). The overall distribution of CNV‐like scores was shifted upward in tumor epithelial cells relative to normal epithelial cells, supporting the presence of broad expression‐based chromosomal deviation in the tumor compartment (Supporting Information 2: Figure S2C). Average chromosome‐level profiles and representative CNV‐like heatmaps further showed that high CNV‐like epithelial cells carried broad and coordinated chromosomal deviation patterns, whereas reference normal cells remained largely flat and CNV‐like‐low cells displayed only limited deviation (Supporting Information 2: Figure S2D,E). Based on this framework, epithelial cells were classified as reference normal, tumor CNV‐like‐low, tumor candidate, or tumor high‐confidence groups. These classes showed distinct spatial distributions within the epithelial UMAP, with high‐confidence malignant cells occupying a relatively concentrated tumor‐associated region (Supporting Information 2: Figure S2F). Subtype‐level annotation further showed that epithelial states differed in their tumor fraction and malignant cell enrichment, and the composition of CNV‐like‐defined epithelial classes varied considerably across tumor samples, indicating substantial intersample heterogeneity in the malignant epithelial compartment (Supporting Information 2: Figure S2G,H,I). We then compared tumor cells with nontumor epithelial populations at both the cell level and the tumor‐only pseudobulk level. Differential expression analysis identified a broad transcriptional shift in tumor epithelial cells, and a consistent set of robust tumor‐associated markers remained elevated when tumor cells were compared with tumor CNV‐like‐low epithelial cells (Supporting Information 2: Figure S2J,K). Functional enrichment showed that malignant epithelial cells were characterized by programs related to MYC targets, oxidative phosphorylation, glycolysis, hypoxia, and reactive oxygen species, together with protein processing and membrane‐associated metabolic functions (Supporting Information 2: Figure S2L,M). Signature analysis further indicated that tumor cells showed higher cell cycle, DNA repair, hypoxia, proliferation, gastric tumor program, and stemness scores than reference normal and CNV‐like‐low epithelial cells, whereas EMT showed a more nuanced pattern and was not the dominant distinguishing feature across all malignant states (Supporting Information 2: Figure S2N,O). Representative tumor‐associated markers were selectively enriched in malignant epithelial cells and showed coherent sample‐level expression patterns across tumor‐enriched epithelial groups (Supporting Information 2: Figure S2P,Q). These data support the presence of transcriptionally and genomically distinct malignant epithelial states in gastric cancer and provide a refined epithelial framework for subsequent analyses of stromal coupling and resistance‐associated programs.

### 3.4. Fibroblasts Occupied a Central Position in Intercellular Communication and Showed Tumor‐Associated Functional Reprogramming

To evaluate the role of fibroblasts in the gastric cancer microenvironment, we performed CellChat analysis after balanced downsampling, which largely preserved the overall microenvironmental structure while improving computational comparability across cell groups (Supporting Information 3: Figure S3A). At the global level, fibroblasts showed extensive communication with multiple compartments, and interactions involving epithelial states were among the most prominent in both number and overall strength (Supporting Information 3: Figure S3B,C). Centrality analysis further placed fibroblasts in a strongly outgoing position within the network, whereas tumor epithelial cells also occupied an active communication state, consistent with reciprocal stromal–epithelial signaling as a major feature of the gastric cancer ecosystem (Supporting Information 3: Figure S3D). Fibroblast‐derived outgoing signals were strongest toward tumor epithelial cells, exceeding those directed to nontumor or normal epithelial states (Supporting Information 3: Figure S3E). When summarized across partners, fibroblast‐centered communication remained particularly strong with tumor epithelial cells, myeloid cells, CD4+ T cells, and mural cells, indicating that fibroblasts were functionally embedded within both stromal and immune interaction circuits (Supporting Information 3: Figure S3F). Pathway‐level analysis showed that fibroblast‐associated signaling toward tumor epithelial cells was dominated by matrix and adhesion‐related pathways, particularly collagen, THBS, FN1, laminin, and MIF (Supporting Information 3: Figure S3G). In the reverse direction, epithelial‐to‐fibroblast communication was comparatively more restricted and involved axes such as MIF–ACKR3, MIF–CD74/CD44, and laminin family interactions with CD44 (Supporting Information 3: Figure S3H). Ligand–receptor mapping further confirmed that fibroblast‐to‐epithelial communication was enriched for collagen‐ and laminin‐centered interactions, whereas epithelial‐to‐fibroblast signaling remained more limited (Supporting Information 3: Figure S3I,J,K). At the transcriptional level, tumor‐associated fibroblasts showed differential expression characterized by increased TAGLN and TPM2 and reduced SLPI, PI16, TNXB, and CFD (Supporting Information 3: Figure S3L). Enrichment analysis linked these cells to extracellular matrix remodeling, MYC targets, interferon‐alpha response, oxidative phosphorylation, and related biosynthetic programs (Supporting Information 3: Figure S3M). UMAP‐based scoring further showed that fibroblast‐associated programs were unevenly distributed across the compartment, with ECM remodeling, contractile myofibroblast‐like features, inflammatory CAF‐like programs, and quiescent fibroblast states occupying distinct regions (Supporting Information 3: Figure S3N). This pattern supports a central role for fibroblasts as communication hubs with tumor‐associated transcriptional reprogramming.

### 3.5. Fibroblast Reclustering Revealed Distinct CAF Subtypes and Highlighted IGF1+ CXCL12+ CAFs as the Subtype Most Enriched for Resistance‐Related Programs

To further resolve stromal heterogeneity, we reclustered the fibroblast compartment and identified five transcriptionally distinct fibroblast/CAF subtypes (Figure 2A). Based on marker expression and overall transcriptional features, these clusters were annotated as resident fibroblasts, IGF1+ CXCL12+ CAFs, HAS1+ inflammatory CAFs, POSTN+ matrix CAFs, and ASPN+ desmoplastic CAFs (Figure 2B). Their relative abundance differed substantially between normal and tumor tissues. Resident fibroblasts accounted for a larger fraction of normal tissues, whereas POSTN+ matrix CAFs and ASPN+ desmoplastic CAFs were expanded in tumors. IGF1+ CXCL12+ CAFs also showed higher representation in tumors, whereas HAS1+ inflammatory CAFs tended to decrease in the tumor compartment (Figure 2C,D). Marker mapping further supported these subtype annotations, with resident fibroblasts enriched for CFD, PI16, and C7; IGF1+ CXCL12+ CAFs marked by IGF1, CXCL12, and CCDC80; POSTN+ matrix CAFs characterized by POSTN, CTSC, and APOE; and ASPN+ desmoplastic CAFs enriched for COL1A1, COL3A1, THBS1, and SPARCL1 (Figure 2E). Functionally, the IGF1+ CXCL12+ CAF state showed inflammatory and secretory features overlapping partly with iCAF‐like programs, whereas POSTN+ matrix CAFs and ASPN+ desmoplastic CAFs were closer to matrix‐remodeling or myofibroblast‐like biology. These categories were therefore used as dataset‐specific CAF states rather than as one‐to‐one replacements for canonical CAF classes. Subtype‐versus‐rest enrichment suggested that IGF1+ CXCL12+ CAFs were linked to inflammatory signaling, TNF‐A/NF‐*κ*B activity, wound healing, and survival‐associated programs, whereas POSTN+ matrix CAFs and ASPN+ desmoplastic CAFs were more strongly associated with ECM remodeling, TGF‐beta signaling, angiogenesis, and focal adhesion‐related features (Figure 2F–H). When compared against previously reported resistance‐associated CAF programs, IGF1+ CXCL12+ CAFs showed the strongest similarity to the IGF1 CAF signature and also scored prominently for PPIC‐labeled and C3 complement CAF‐related programs (Figure 2I–K). Tumor sample‐level analysis supported this pattern, with resistance‐related CAF signatures preferentially projecting onto IGF1+ CXCL12+ CAFs across multiple tumor samples (Figure 2L). In parallel, subtype‐level coupling analysis suggested that different fibroblast states were linked to distinct tumor microenvironmental features, whereas communication analysis showed that all fibroblast subtypes signaled most strongly toward tumor epithelial cells, with collagen‐centered signaling particularly prominent across activated CAF states (Figure 2M–P). These data indicate that gastric cancer fibroblasts comprise multiple biologically distinct CAF states, among which IGF1+ CXCL12+ CAFs most consistently concentrate known resistance‐supportive signatures.

Figure 2Fibroblast reclustering reveals distinct CAF subtypes and highlights IGF1+ CXCL12+ CAFs as the subtype most enriched for resistance‐related programs. (A) UMAP of fibroblasts colored by unsupervised cluster identity. (B) UMAP showing annotation of fibroblast/CAF subtypes, including resident fibroblasts, IGF1+ CXCL12+ CAFs, HAS1+ inflammatory CAFs, POSTN+ matrix CAFs, and ASPN+ desmoplastic CAFs. (C) Relative composition of fibroblast subtypes in normal and tumor tissues. (D) Paired comparison of patient‐level fibroblast subtype proportions between normal and tumor tissues. (E) Marker landscape across fibroblast subtypes. Dot size indicates the fraction of expressing cells, and color intensity indicates average expression level. (F) Heatmap showing subtype‐versus‐rest enrichment of representative functional programs. (G) UMAP overlays of representative fibroblast‐related programs, including EMT, TGF‐beta signaling, ECM collagen remodeling, and hypoxia. (H) Heatmap showing functional program activity across fibroblast subtypes. (I) Heatmap showing subtype‐level similarity to known resistance‐associated CAF programs. (J) UMAP overlays of representative CAF signatures, including IGF1 CAF, PPIC‐labeled CAF, and C3 complement CAF signatures. (K) Violin plots showing resistance‐associated CAF signature scores across fibroblast subtypes. (L) Tumor sample average resistance‐related CAF signature scores across fibroblast subtypes. (M) Representative subtype‐level coupling relationships across tumor samples. (N) Outgoing communication strength from fibroblast subtypes to major target cell groups. (O) Fibroblast subtype activity across selected ligand–receptor signaling axes. (P) Top ligand–receptor pairs linking fibroblast subtypes to key target cell populations.

**Figure:**
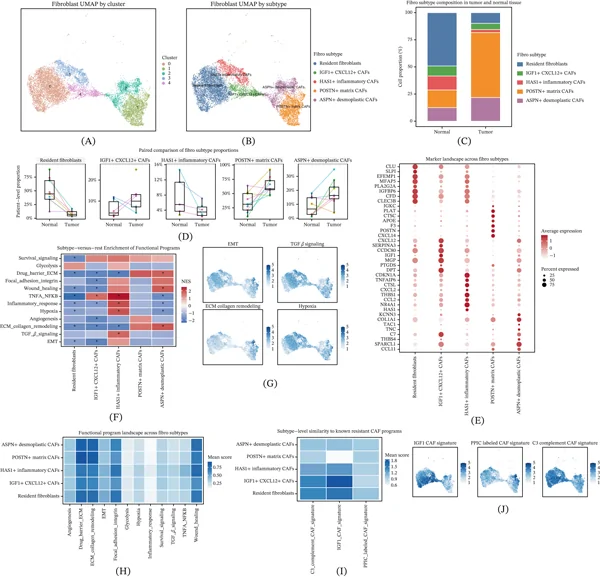
(a)

**Figure:**
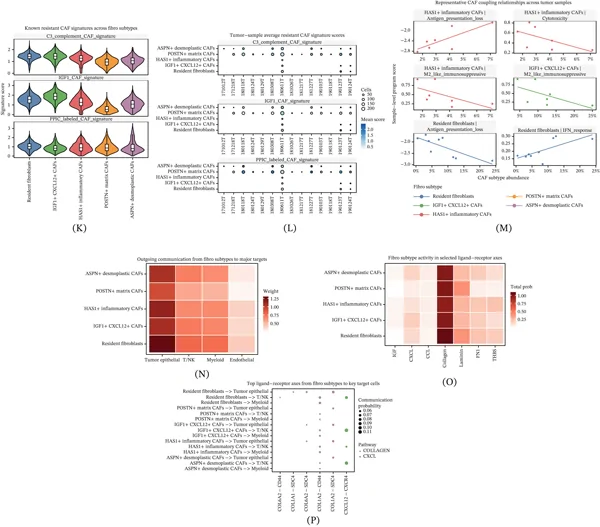
(b)

### 3.6. IGF1+ CXCL12+ CAFs Displayed a Concentrated Resistance‐Supportive Phenotype

We next examined the molecular and functional features of IGF1+ CXCL12+ CAFs in greater detail. This subtype formed a distinct region within the fibroblast landscape and was readily separated from the remaining fibroblast populations (Supporting Information 4: Figure S4A). Feature mapping confirmed selective enrichment of IGF1, CXCL12, IGFBP4, SFRP2, DPT, and CCDC80 within this compartment, together with additional stromal markers such as C3, CFD, and FBLN1 (Supporting Information 4: Figure S4B). Differential expression analysis against the remaining fibroblasts further supported its distinct transcriptional identity, with upregulated genes centered on stromal signaling and secretory features, whereas genes such as POSTN, COL1A1, and COL3A1 were relatively reduced compared with matrix‐dominant CAF states (Supporting Information 4: Figure S4C). Within the IGF1+ CXCL12+ CAF compartment itself, tumor‐associated cells also showed additional transcriptional shifts relative to their normal counterparts, indicating that this subtype was not only present across tissues but also underwent tumor‐related reprogramming (Supporting Information 4: Figure S4D). Functionally, IGF1+ CXCL12+ CAFs were enriched for multiple resistance‐supportive programs. Enrichment analysis highlighted pathways related to stress adaptation, wound response, EMT‐associated support, and survival‐related signaling, whereas tumor‐versus‐normal comparison within this subtype pointed to additional shifts linked to stress response and microenvironmental adaptation (Supporting Information 4: Figure S4E,F). Signature comparisons showed that IGF1+ CXCL12+ CAFs scored prominently for CXCL12 retention, drug barrier ECM, IGF1 CAF signature, resistance support index, and TGF‐beta signaling, although these programs were not uniformly exclusive to this subtype (Supporting Information 4: Figure S4G,H). At the abundance level, IGF1+ CXCL12+ CAFs tended to be more represented in tumors than in paired normal tissues, but the magnitude of increase varied across samples (Supporting Information 4: Figure S4I). Sample‐level heatmaps further showed that this subtype retained a relatively coherent functional profile across tumors despite intersample heterogeneity, particularly for IGF1‐axis, resistance support, hypoxia, glycolysis, and ECM‐related features (Supporting Information 4: Figure S4J). Marker‐level summaries at both the subtype and sample levels reinforced the selective expression of the IGF1+ CXCL12+ CAF program within this compartment (Supporting Information 4: Figure S4K,L). These observations support prioritizing IGF1+ CXCL12+ CAFs for subsequent coexpression and stromal–epithelial coupling analyses.

### 3.7. External CF Chemotherapy Bulk Validation Supported an Increase in IGF1+ CXCL12+ CAF‐Related Programs

To assess whether this stromal program was detectable in an independent chemotherapy‐related setting, we analyzed the paired GSE14209 bulk cohort of gastric cancer biopsies collected before and after CF chemotherapy. Signature‐level paired analysis showed heterogeneous treatment‐associated changes across patients. The C3_complement_CAF signature and several CAF‐related scores showed upward trends after treatment, whereas paired tests for the predefined CAF signatures did not remain significant after multiple‐testing correction (Supporting Information 5: Figure S5A,B,F). At the pathway level, ranked gene enrichment analysis revealed treatment‐associated changes in inflammatory, hypoxia, EMT, angiogenesis, and stromal remodeling programs, including TNF‐A/NF‐*κ*B signaling, inflammatory response, interferon‐gamma response, IL‐6/JAK/STAT3 signaling, ECM remodeling, fibroblast activation, and an immunomodulation‐related custom signature (Supporting Information 5: Figure S5C). Patient‐level heatmap analysis further indicated substantial interpatient heterogeneity, with selected IGF1+ CXCL12+ CAF‐related scores increasing in a subset of cases (Supporting Information 5: Figure S5D). The running score plot for the IGF1+ CXCL12+ CAF core signature was directionally consistent with the broader pathway analysis, although the enrichment strength was modest (Supporting Information 5: Figure S5E). IGF1 and CXCL12 expression showed only a modest inverse correlation in the bulk cohort, suggesting that the posttreatment signal likely reflected a broader stromal program rather than strict gene‐level coexpression (Supporting Information 5: Figure S5G). These external data therefore provide supportive, pathway‐level evidence that CF chemotherapy is accompanied by remodeling of selected stromal and inflammatory programs, while not constituting definitive validation of a single CAF signature or gene.

### 3.8. hdWGCNA Identified Resistance‐Associated Coexpression Modules Within IGF1+ CXCL12+ CAFs

**Figure 3:**
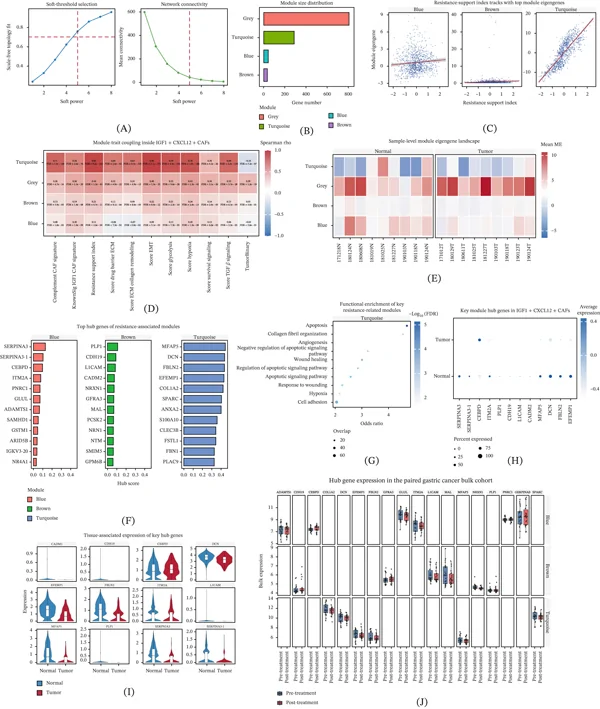
hdWGCNA identifies resistance‐associated coexpression modules within IGF1+ CXCL12+ CAFs. (A) Soft‐thresholding selection and network topology analysis for hdWGCNA. (B) Distribution of detected module sizes. (C) Association between the resistance support index and eigengenes of the major modules. (D) Heatmap showing module–trait correlations within IGF1+ CXCL12+ CAFs. (E) Sample‐level landscape of module eigengenes in normal and tumor samples. (F) Top hub genes from representative resistance‐associated modules. (G) Functional enrichment of representative resistance‐related modules. (H) Dot plot showing representative module hub genes in IGF1+ CXCL12+ CAFs. (I) Tissue‐associated expression patterns of representative hub genes in IGF1+ CXCL12+ CAFs. (J) Expression of representative hub genes in the paired gastric cancer bulk cohort before and after treatment.

To further resolve the internal regulatory structure of IGF1+ CXCL12+ CAFs, we performed hdWGCNA within this subtype. Soft‐thresholding analysis supported network construction under a scale‐free topology‐compatible setting, and module detection identified a limited number of coexpression modules, among which the turquoise module was the largest, followed by smaller blue and brown modules (Figure 3A,B). Among these, the turquoise module showed the clearest positive relationship with the resistance support index, whereas the blue and brown modules displayed weaker or more restricted associations (Figure 3C). Module–trait correlation analysis further showed that the turquoise module tracked not only the composite resistance support score but also multiple related programs, including hypoxia, glycolysis, survival signaling, drug barrier ECM, and known IGF1‐associated CAF signatures, indicating that this module captured a broader resistance‐supportive stromal state rather than a single isolated pathway (Figure 3D). At the sample level, module eigengene patterns were more pronounced in tumor samples than in normal samples, particularly for the turquoise module, whereas the blue and brown modules remained more modest (Figure 3E). Hub‐gene analysis highlighted distinct gene sets across the resistance‐associated modules. The blue module contained genes such as SERPINA3, SERPINA3‐1, CEBPD, and ITM2A; the brown module included PLP1, CDH19, ICAM1, and CADM2; and the turquoise module was enriched for stromal structural and matrix‐related genes, including MFAP5, DCN, FBLN2, EFEMP1, COL1A2, and SPARC (Figure 3F). Functional enrichment analysis pointed to processes related to collagen fibril organization, angiogenesis, wound healing, regulation of apoptotic signaling, and cell adhesion, consistent with a stromal program capable of supporting adaptation and persistence under treatment pressure (Figure 3G). Dot plot visualization showed that representative hub genes were preferentially detectable within IGF1+ CXCL12+ CAFs, although their expression levels varied between tumor and normal contexts (Figure 3H,I). In the external paired bulk cohort, several hub genes remained measurable and showed heterogeneous but directionally interpretable treatment‐associated changes, suggesting that the module‐level signals derived from single‐cell data were at least partially recoverable in independent transcriptomic data (Figure 3J). This analysis indicates that IGF1+ CXCL12+ CAFs contain an internally organized coexpression structure, with one dominant resistance‐associated module linking matrix remodeling, stress adaptation, and stromal support functions.

### 3.9. CAF‐Epithelial Coupling Analysis Identified ARPC1A as a Candidate Gene Linked to the CAF Resistance Axis

To examine whether the resistance‐supportive stromal state was associated with epithelial programs in tumor cells, we integrated IGF1+ CAF abundance, module‐derived CAF projections, hub‐gene projections, and known IGF1‐related stromal signals to generate a sample‐level CAF resistance axis. Across shared tumor samples, these CAF‐side variables showed coordinated variation, although the magnitude differed between patients (Figure 4A). Correlation analysis further showed that the CAF resistance axis and related stromal indicators were positively associated with several epithelial programs, particularly hypoxia, partial EMT, TGF‐beta signaling, survival‐related features, and the gastric tumor program, whereas associations with DNA repair and angiogenesis were weaker or more variable (Figure 4B). These findings indicate that the stromal state defined by IGF1+ CXCL12+ CAF‐associated signals was linked to a distinct set of adaptive epithelial programs rather than to a single uniform malignant phenotype. At the gene level, correlation analysis identified a subset of tumor epithelial genes positively associated with the CAF resistance axis (Figure 4C). Enrichment analysis suggested that these CAF‐coupled epithelial genes were mainly related to extracellular matrix interaction, cell adhesion, antiapoptotic signaling, wound response, and selected stress adaptation pathways (Figure 4D). Their sample‐level expression patterns were also broadly concordant across tumor samples with higher CAF resistance scores (Figure 4E). Representative genes, including CD24, SYNGR2, RHOD, ARPC1A, CD55, and ALDH2, were preferentially detected in tumor epithelial cells on UMAP projection rather than in reference normal or CNV‐like‐low epithelial states (Figure 4F,G). Regression‐based prioritization further supported ARPC1A as a stable and experimentally tractable candidate within the CAF‐coupled epithelial set (Figure 4H). Specifically, ARPC1A combined positive association with the CAF resistance axis, concordance with the epithelial resistance index, preferential tumor epithelial expression, and supportive TCGA expression and survival patterns, while also having a clear loss‐of‐function validation strategy in gastric cancer cells. We next assessed the clinical relevance of ARPC1A in TCGA‐STAD. Higher ARPC1A expression was associated with shorter overall survival, and ARPC1A expression was higher in tumor tissues than in normal gastric tissues (Figure 4I,J). These results suggest that CAF‐associated stromal remodeling is linked to a distinct epithelial response program and highlight ARPC1A as a candidate epithelial gene associated with this program. Because the study was intended to connect microenvironmental remodeling with copy‐number and genomic alteration context, we next examined how ARPC1A is positioned within the TCGA‐STAD genomic landscape.

Figure 4CAF‐epithelial coupling analysis identifies ARPC1A as a candidate epithelial gene associated with the CAF resistance program. (A) Heatmap showing the CAF resistance axis and its component stromal features across shared tumor samples. (B) Heatmap showing correlations between CAF‐side indicators and epithelial programs. (C) Plot showing tumor epithelial genes associated with the CAF resistance axis. (D) Enrichment analysis of CAF‐coupled tumor epithelial genes. (E) Heatmap showing sample‐level expression patterns of representative CAF‐coupled epithelial candidate genes. (F) UMAP feature plots showing representative candidate genes in epithelial cells. (G) Dot plot showing candidate gene expression across epithelial states. (H) Coefficient path plot used for candidate gene prioritization. (I) Kaplan–Meier overall survival curve for ARPC1A in TCGA‐STAD. (J) Comparison of ARPC1A expression between normal and tumor tissues in TCGA‐STAD. The asterisks indicate statistical significance: ^*^ *p* < 0.05; ^**^ *p* < 0.01.

**Figure:**
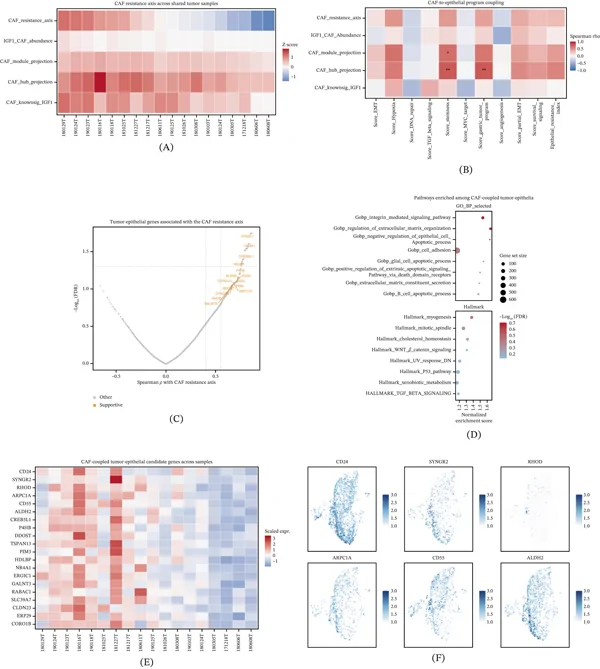
(a)

**Figure:**
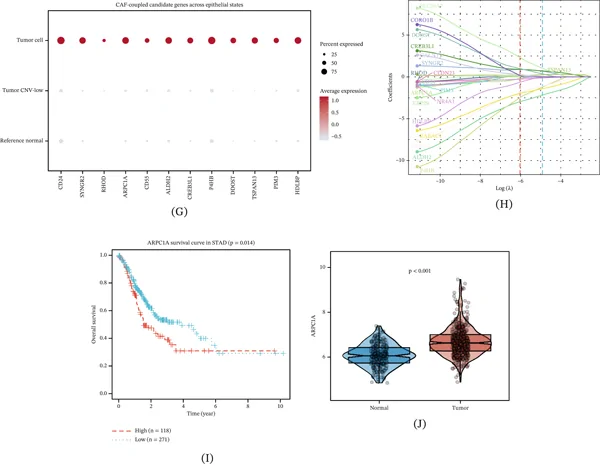
(b)

### 3.10. TCGA‐STAD Genomic Contextualization and DepMap CRISPR Virtual Knockout Analysis of ARPC1A

**Figure 5:**
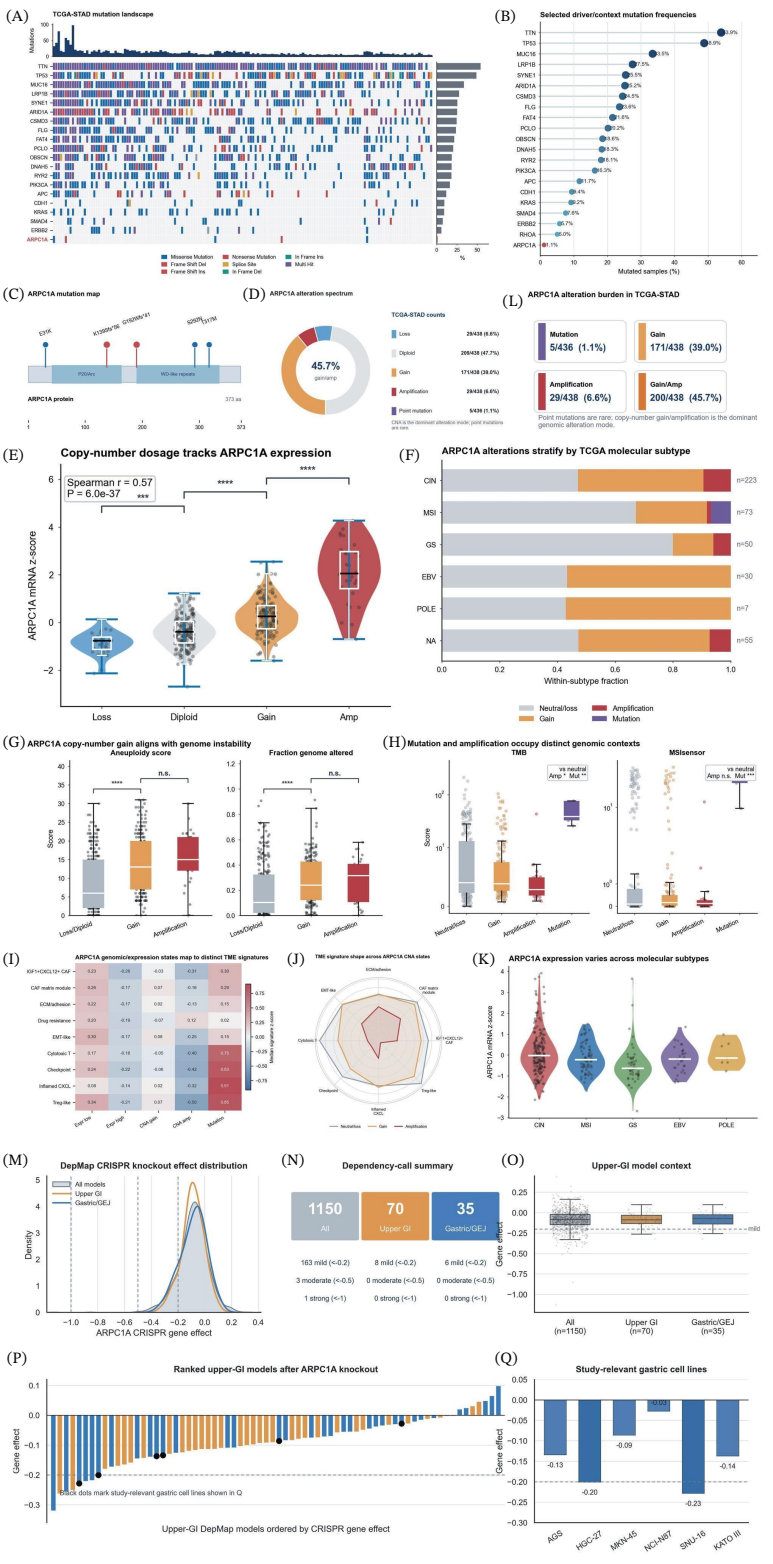
TCGA‐STAD mutation/copy‐number and DepMap CRISPR virtual knockout analyses contextualize ARPC1A as a rare mutant but copy‐number‐enriched candidate with weak‐to‐mild dependency effects in gastric cancer models. (A) Oncoplot showing the mutation landscape of selected gastric cancer driver and context genes in TCGA‐STAD, with ARPC1A included as the CAF‐linked candidate gene. (B) Lollipop‐style frequency plot showing mutation frequencies of selected genes. (C) ARPC1A protein‐domain mutation map showing the positions and types of ARPC1A variants detected in TCGA‐STAD, including missense and frameshift events. (D) Donut and count summary showing ARPC1A copy‐number loss, diploid status, gain, amplification, and mutation frequencies. (E) Violin and box plots showing the relationship between ARPC1A copy‐number status and ARPC1A mRNA *z*‐score, with Spearman correlation and statistical comparisons. (F) TCGA molecular subtype distribution across ARPC1A alteration states. (G) Comparison of aneuploidy score and fraction of genome altered across ARPC1A copy‐number groups. (H) TMB and MSIsensor score distributions across neutral/loss, gain, amplification, and mutation groups. (I) Heatmap showing median CAF‐related, EMT‐like, drug‐resistance, and immune signature scores across ARPC1A genomic and expression states. (J) Radar plot summarizing selected tumor microenvironment signature patterns across ARPC1A genomic states. (K) ARPC1A expression distribution across TCGA molecular subtypes. (L) Summary tiles showing the burden of ARPC1A mutation, gain, amplification, and combined gain/amplification in TCGA‐STAD. (M) DepMap Public 24Q2 CRISPR gene‐effect distribution for ARPC1A across all screened models, upper GI models, and gastric/GEJ models. More negative gene‐effect values indicate stronger fitness loss after CRISPR knockout. (N) Dependency call summary across all screened, upper GI, and gastric/GEJ model groups using standard DepMap thresholds. (O) Box and point plots comparing ARPC1A CRISPR gene‐effect distributions across all screened models, upper GI models, and gastric/GEJ models. (P) Ranked ARPC1A CRISPR gene‐effect values across upper GI models, with black dots marking study‐relevant gastric cancer cell lines shown in Figure 5Q. (Q) Highlighted ARPC1A CRISPR gene‐effect scores for selected gastric cancer cell lines used or referenced in this study. DepMap Public 24Q2 was used as the CRISPR virtual knockout source. The asterisks indicate statistical significance: ^*^ *p* < 0.05, ^**^ *p* < 0.01, ^***^ *p* < 0.001, and ^* * * *^p < 0.0001.

To determine whether ARPC1A is primarily altered through point mutation or broader genomic dosage changes in gastric cancer, we analyzed the TCGA‐STAD Pan‐Cancer Atlas cohort. The overall mutation landscape showed high mutation frequencies in established gastric cancer context genes, including TTN, TP53, MUC16, LRP1B, SYNE1, ARID1A, CSMD3, and FLG, whereas ARPC1A mutations were infrequent (5/436 sequenced samples, 1.1%) (Figure 5A,B). Mapping of ARPC1A variants onto the protein sequence showed three missense variants (E31K, S292N, and T317M) and two frameshift truncating variants (K139Sfs∗56 and G192Wfs∗41), without a recurrent hotspot (Figure 5C). This distribution does not support a recurrent gain‐of‐function hotspot mechanism. The two frameshift mutations are compatible with loss of function, whereas the three scattered missense variants remain variants of uncertain functional consequence in the absence of allele‐specific assays. In contrast, ARPC1A copy‐number gain or amplification was substantially more common than point mutation, with gain/amplification detected in 200/438 samples (45.7%) and focal amplification in 29/438 samples (6.6%) (Figure 5D). Copy‐number status was strongly associated with ARPC1A mRNA abundance, with progressively higher expression from loss and diploid states to gain and amplification (Spearman *r* = 0.57, *p* = 6.0e − 37) (Figure 5E). Molecular subtype stratification further indicated that ARPC1A amplification was enriched mainly in chromosomal instability tumors, whereas all five ARPC1A‐mutant cases occurred in MSI tumors (Figure 5F). Consistently, ARPC1A‐mutant tumors showed markedly higher TMB and MSIsensor scores than amplification‐driven cases (median TMB, 38.77 vs. 2.85 mutations/Mb; median MSIsensor score, 34.35 vs. 0.13), whereas amplification‐driven cases showed higher aneuploidy and fraction genome altered (median fraction genome altered, 0.316 vs. 0.061) (Figure 5G,H). We next compared mutation‐associated and amplification‐associated phenotypes using tumor‐level signatures. Compared with amplification‐driven cases, ARPC1A‐mutant tumors showed higher cytotoxic T‐cell, checkpoint, inflamed CXCL, and Treg‐like signatures, consistent with their MSI/high TMB immune context (Figure 5I,J). By contrast, CAF‐related scores, drug‐resistance score, and EMT‐like score did not show a stable mutation‐specific increase relative to amplification‐driven cases. Direct cisplatin response information was not available in TCGA‐STAD; therefore, drug‐resistance and EMT‐like transcriptomic scores were used as indirect phenotype readouts rather than clinical response endpoints. ARPC1A expression also varied across TCGA molecular subtypes, supporting the interpretation that ARPC1A is embedded in subtype‐specific genomic and transcriptional contexts rather than functioning as an isolated candidate gene (Figure 5K,L). These observations position the TCGA analysis as a mutation‐phenotype and copy‐number contextualization of a CAF‐associated epithelial candidate. They suggest that ARPC1A‐mutant tumors are best interpreted as rare MSI/high TMB tumors with immune‐activated microenvironmental features, whereas ARPC1A gain/amplification represents a more common expression‐dosage mechanism. To complement this genomic landscape with a functional dependency estimate, we analyzed DepMap Public 24Q2 CRISPR gene‐effect data for ARPC1A as a virtual knockout resource. Across all screened models (*n* = 1150), ARPC1A depletion produced a median gene‐effect score of −0.081, with 163 mild, 3 moderate, and 1 strong dependency calls using standard DepMap thresholds (Figure 5M,N). Upper GI models showed a similar distribution (*n* = 70, median = −0.088), with eight mild and no moderate or strong dependencies, whereas gastric/GEJ models (*n* = 35, median = −0.071) contained six mild dependency calls and no moderate or strong dependencies (Figure 5N,O). Ranking of upper GI models showed that only a subset approached or crossed the mild dependency threshold and study‐relevant gastric cancer cell lines displayed weak‐to‐mild ARPC1A gene‐effect scores, including AGS (−0.13), HGC‐27 (−0.20), MKN‐45 (−0.09), NCI‐N87 (−0.03), SNU‐16 (−0.23), and KATO III (−0.14) (Figure 5P,Q). Together, these analyses indicate that ARPC1A is unlikely to represent a recurrent gain‐of‐function mutation driver or a strong pan‐essential survival gene in gastric cancer models. Instead, the combined TCGA and DepMap data support a model in which rare ARPC1A mutations occur mainly in MSI/high TMB tumors, whereas copy‐number gain/amplification and expression dosage provide the more prominent genomic context for the CAF‐associated ARPC1A program.

### 3.11. ARPC1A Promoted Malignant Phenotypes and Cisplatin Tolerance in Gastric Cancer Cells

**Figure 6:**
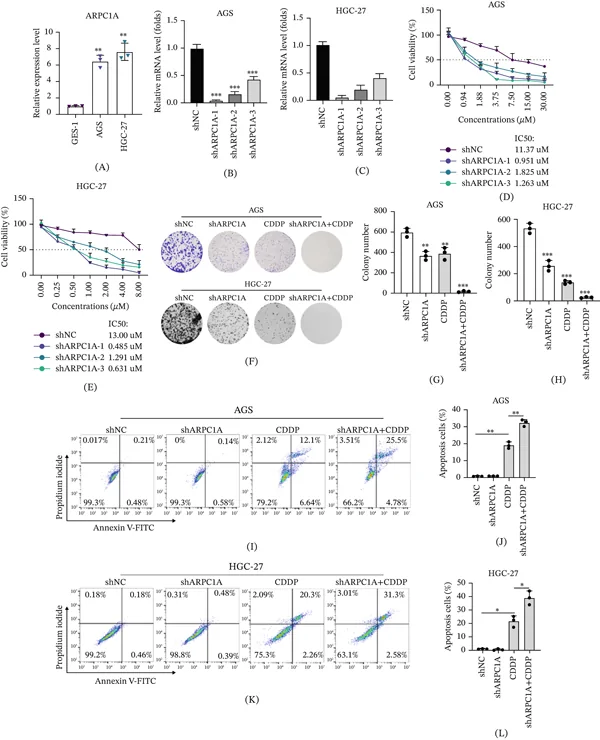
ARPC1A is upregulated in gastric cancer cells, and its silencing enhances cisplatin sensitivity while suppressing clonogenic survival. (A) qRT‐PCR analysis of ARPC1A mRNA expression in gastric cancer cell lines (AGS and HGC‐27) and the normal gastric epithelial cell line (GES‐1). (B, C) qRT‐PCR validation of ARPC1A knockdown efficiency in AGS and HGC‐27 cells transduced with three independent shRNAs (shARPC1A‐1, shARPC1A‐2, and shARPC1A‐3) or negative control (shNC). (D, E) CCK‐8 assay showing cisplatin dose–response curves and calculated IC50 values in AGS and HGC‐27 cells with stable ARPC1A knockdown or control cells. (F) Representative images of colony formation assays in AGS and HGC‐27 cells under the indicated conditions (shNC, shARPC1A, cisplatin, and shARPC1A + cisplatin). (G, H) Quantification of colony numbers in AGS and HGC‐27 cells under the indicated conditions. (I, K) Representative flow cytometry plots of Annexin V‐FITC/PI staining in AGS and HGC‐27 cells under the indicated conditions. (J, L) Quantification of apoptotic cells in AGS and HGC‐27 cells under the indicated conditions. CDDP in the figure denotes cisplatin. Data are presented as mean ± SD from three independent experiments.  ^∗^ *p* < 0.05,  ^∗∗^ *p* < 0.01, and  ^∗∗∗^ *p* < 0.001.

Given that ARPC1A emerged from the CAF‐epithelial coupling analysis as a candidate epithelial gene associated with the CAF resistance axis, we performed functional validation in gastric cancer cell lines. qRT‐PCR showed that ARPC1A expression was higher in AGS and HGC‐27 cells than in the normal gastric epithelial cell line GES‐1, supporting its upregulation in gastric cancer cells (Figure 6A). Stable knockdown models were then established using three independent shRNAs targeting ARPC1A. In both AGS and HGC‐27 cells, all three shRNAs reduced ARPC1A mRNA expression, with shARPC1A‐1 showing the strongest silencing efficiency and therefore being selected for subsequent assays (Figure 6B,C). We next examined whether ARPC1A influences cisplatin response. CCK‐8 assays showed that stable ARPC1A knockdown reduced the IC50 of cisplatin in both AGS and HGC‐27 cells, indicating increased cisplatin sensitivity after ARPC1A depletion (Figure 6D,E). Consistent with this finding, colony formation assays showed that ARPC1A knockdown alone suppressed clonogenic growth, whereas the combination of ARPC1A silencing and cisplatin treatment produced the strongest inhibitory effect on colony formation in both cell lines (Figure 6F,H). To determine whether increased cisplatin sensitivity was accompanied by enhanced apoptosis, we performed Annexin V‐FITC/PI flow cytometry. In both AGS and HGC‐27 cells, ARPC1A knockdown alone had little effect on basal apoptosis, whereas cisplatin treatment increased the proportion of apoptotic cells. The combination of ARPC1A knockdown and cisplatin further increased apoptosis compared with cisplatin treatment alone (Figure 6I,L). These results indicate that ARPC1A supports clonogenic survival and cisplatin tolerance in gastric cancer cells and that its depletion sensitizes gastric cancer cells to cisplatin‐induced apoptosis.

### 3.12. ARPC1A Knockdown Suppressed Migratory and Invasive Capacities of Gastric Cancer Cells

We next examined whether ARPC1A contributes to the migratory and invasive phenotypes of gastric cancer cells. In wound‐healing assays, ARPC1A knockdown delayed wound closure in both AGS and HGC‐27 cells compared with the corresponding shNC controls (Supporting Information 6: Figure S6A). Quantification showed that migration rates were reduced after ARPC1A silencing in both cell lines, indicating that ARPC1A supports the migratory capacity of gastric cancer cells (Supporting Information 6: Figure S6B,C). We further assessed cell invasion using Matrigel‐coated Transwell chambers. Representative images showed fewer invading cells in all three ARPC1A‐silenced groups than in the control group in both AGS and HGC‐27 cells (Supporting Information 6: Figure S6D). Quantitative analysis confirmed that ARPC1A knockdown reduced invasive capacity in both cell lines (Supporting Information 6: Figure S6E,F). Taken together, these results indicate that ARPC1A promotes aggressive phenotypes of gastric cancer cells by supporting migration and invasion.

### 3.13. ARPC1A Knockdown Reduced Expression of Drug‐Resistance and Antiapoptotic Genes While Attenuating EMT‐Related Features

To explore the molecular basis by which ARPC1A knockdown enhanced cisplatin sensitivity, we examined representative drug‐resistance‐ and apoptosis‐related genes by qRT‐PCR. In both AGS and HGC‐27 cells, ARPC1A silencing reduced ABCB1 and ABCC1, two genes associated with drug efflux and chemoresistance. In parallel, the antiapoptotic genes BCL2 and MCL1 were also downregulated after ARPC1A knockdown in both cell lines (Supporting Information 7: Figure S7A,B). These results are consistent with the CCK‐8 and apoptosis assays and support a role for ARPC1A in maintaining a cisplatin‐tolerant state in gastric cancer cells. We next examined EMT‐related gene expression. In both AGS and HGC‐27 cells, ARPC1A silencing increased the epithelial marker CDH1 and reduced the mesenchymal marker VIM (Supporting Information 7: Figure S7C,D). These changes are consistent with the reduced migration and invasion observed after ARPC1A knockdown and suggest that ARPC1A contributes to aggressive phenotypes, at least in part, by supporting EMT‐like transcriptional features. Taken together, these data indicate that ARPC1A promotes both chemoresistance‐associated and EMT‐related programs in gastric cancer cells.

## 4. Discussion

Cisplatin resistance in gastric cancer is often discussed as a tumor cell–intrinsic phenomenon, yet the present study indicates that resistance‐related adaptation is more appropriately understood at the level of the tumor ecosystem [30, 31]. By integrating single‐cell transcriptomics with stromal reclustering, communication analysis, coexpression network modeling, exploratory external treatment‐cohort evaluation, genomic contextualization, and functional experiments, we identified a stromal state centered on IGF1+ CXCL12+ CAFs and associated this state with an epithelial candidate, ARPC1A, that promotes cisplatin tolerance and aggressive phenotypes in gastric cancer cells. Conceptually, this framework extends the interpretation of chemotherapy resistance beyond isolated malignant cell programs and places stromal–epithelial coupling within the broader context of therapeutic adaptation [32, 33].

A major finding of this study is that fibroblast heterogeneity in gastric cancer is not random with respect to resistance‐associated biology [34]. Among the fibroblast states identified, IGF1+ CXCL12+ CAFs were not defined simply by abundance, but by enrichment for resistance‐supportive signatures, a central position in stromal–epithelial communication, and internal organization into resistance‐associated coexpression modules. This subtype shared selected inflammatory and secretory features with iCAF‐like programs while also carrying IGF1‐axis, CXCL12‐retention, and resistance‐supportive signatures, supporting its use as a dataset‐defined CAF state rather than a simple relabeling of canonical CAF classes. At the same time, our results do not suggest that all resistance‐associated features are exclusive to this population. Instead, the data favor a model in which IGF1+ CXCL12+ CAFs constitute a concentrated stromal carrier of these programs, whereas other activated CAF states may contribute partially or context‐dependently.

ARPC1A is a component functionally linked to the Arp2/3 machinery and has been implicated in actin‐dependent cancer cell migration and invasion in prior studies [35, 36]. Its prioritization in our analysis was based on convergent evidence rather than a single ranking metric: ARPC1A showed association with the CAF resistance axis, concordance with epithelial resistance‐related programs, preferential detection in tumor epithelial cells, supportive TCGA expression and survival patterns, and feasibility for loss‐of‐function validation. Functional experiments then supported the biological relevance of this prioritization. ARPC1A was upregulated in gastric cancer cells, and its depletion reduced cisplatin IC50, impaired colony formation, enhanced cisplatin‐induced apoptosis, and attenuated migration and invasion. In parallel, ARPC1A knockdown reduced ABCB1, ABCC1, BCL2, and MCL1 expression while increasing CDH1 and decreasing VIM, linking ARPC1A to chemoresistance‐associated and EMT‐related transcriptional features [33, 37, 38]. These data support ARPC1A as a functionally relevant epithelial component associated with the stromal resistance program, while not proving that CAF‐derived signals directly induce ARPC1A.

The genomic analysis further clarifies how ARPC1A should be interpreted in gastric cancer and places the study more directly within a mutation‐immune crosstalk framework. ARPC1A did not appear to represent a frequently mutated oncogene in TCGA‐STAD. Its mutation spectrum contained both scattered missense variants and frameshift truncating variants, with no recurrent hotspot. Therefore, the available cohort‐level data do not support a clear ARPC1A gain‐of‐function mutation mechanism. The frameshift variants are compatible with loss of function, but their rarity and MSI/high TMB background make it difficult to assign a direct ARPC1A‐specific phenotype. Importantly, ARPC1A‐mutant tumors showed stronger immune activation signatures than amplification‐driven cases, including cytotoxic T‐cell, checkpoint, inflamed CXCL, and Treg‐like signals, consistent with MSI‐linked mutation‐immune crosstalk. In contrast, CAF‐related, EMT‐like, and drug‐resistance signatures did not separate mutant tumors from amplification‐driven tumors in a stable manner. By contrast, copy‐number gain and amplification were more common and showed a dosage relationship with ARPC1A mRNA expression, as well as enrichment in chromosomal instability tumors and association with genome‐instability metrics. This distinction is important: ARPC1A is more plausibly linked to gastric cancer through expression dosage, subtype context, functional dependency, and stromal–epithelial coupling than through recurrent activating point mutation. Thus, the TCGA and DepMap analyses provide mutation‐immune, mutation‐phenotype, and copy‐number contextualization that refines interpretation of ARPC1A rather than establishing ARPC1A as a mutation‐driven driver gene.

The present findings have pharmacological implications for how platinum response is interpreted in gastric cancer. Rather than treating cisplatin tolerance as a solely tumor cell–intrinsic property, our data support a model in which stromal signaling, extracellular matrix remodeling, and epithelial adaptive programs jointly shape drug response. In this framework, IGF1+ CXCL12+ CAFs represent a resistance‐supportive stromal context, whereas ARPC1A represents an epithelial effector associated with this context. The current data do not prove direct CAF‐mediated induction of ARPC1A or establish that ARPC1A targeting alone would reverse stromal protection in vivo; instead, they provide a prioritized CAF‐associated epithelial candidate that can be tested in coculture, spatial, and in vivo pharmacology models.

Several limitations should be acknowledged. First, although the single‐cell and bulk analyses supported the existence of a resistance‐associated stromal program, the external paired bulk cohort provided supportive rather than definitive validation, and several signature‐level comparisons did not remain significant after multiple‐testing correction. Second, the communication and coupling analyses are inferential and do not prove direct causal signaling between IGF1+ CXCL12+ CAFs and ARPC1A‐expressing epithelial cells. Third, TCGA‐STAD provides bulk tumor genomic data and does not identify ARPC1A‐mutated CAF subpopulations directly; therefore, the mutation‐associated CAF and immune analyses rely on tumor‐level signature scores rather than cell type–resolved mutation calls. Fourth, the ARPC1A‐mutant group was very small, and direct cisplatin response annotations were not available in TCGA‐STAD, limiting mutation‐phenotype analyses to transcriptomic surrogates such as drug‐resistance, EMT‐like, CAF, and immune signatures. Fifth, the TCGA‐STAD genomic analysis provides cohort‐level context for mutation, copy‐number, expression, subtype, and genome‐instability patterns, but it does not establish causality between ARPC1A copy‐number alteration and chemotherapy response. Sixth, the DepMap CRISPR analysis estimates baseline fitness effects in cell line models and should not be interpreted as a direct substitute for drug‐response assays or stromal coculture perturbation. Finally, the functional experiments were performed in vitro in gastric cancer cell lines and did not recapitulate the full stromal and immune complexity of the tumor microenvironment. Future work incorporating allele‐specific ARPC1A‐mutant models, CAF tumor coculture systems, spatial validation, immune‐context analyses, genomic perturbation models, and in vivo treatment models will be important to determine whether resistance‐associated CAF states and ARPC1A‐linked epithelial vulnerabilities can be therapeutically exploited.

Taken together, this study defines an IGF1+ CXCL12+ CAF‐centered stromal state associated with resistance‐supportive biology in gastric cancer and identifies ARPC1A as a CAF‐associated epithelial effector involved in cisplatin tolerance, survival, migration, and invasion. By integrating single‐cell ecosystem analysis, TCGA‐STAD mutation‐immune/copy‐number context, DepMap CRISPR dependency information, and functional validation, we provide a framework in which platinum response is interpreted as an emergent property of stromal–epithelial interaction, mutation‐immune crosstalk, and epithelial adaptation rather than an isolated tumor cell trait. These findings refine our understanding of gastric cancer drug response and support further mechanistic evaluation of ARPC1A and resistance‐associated CAF states as potential entry points for overcoming cisplatin tolerance.

## 5. Conclusion

In summary, our study identifies an IGF1+ CXCL12+ CAF‐centered stromal state associated with cisplatin response biology in gastric cancer and links this state to ARPC1A, a candidate epithelial effector embedded primarily in a mutation‐immune and copy‐number/expression‐dosage genomic context. ARPC1A mutations were rare, mixed in predicted consequence, and confined to MSI tumors; mutation‐associated phenotypes were more consistent with MSI‐linked immune activation and mutation‐immune crosstalk than with a distinct ARPC1A mutation‐specific CAF, EMT, or drug‐resistance state. Functional experiments further showed that ARPC1A depletion enhances cisplatin sensitivity, increases apoptosis, and suppresses clonogenic survival, migration, and invasion. These findings provide a tumor‐ecosystem framework for understanding platinum response in gastric cancer and support further investigation of CAF‐associated epithelial vulnerabilities and allele‐specific ARPC1A mechanisms.

## Author Contributions

D.L., F.M., and W.Z. contributed equally to this work and share the first authorship. D.L., F.M., and W.Z. performed the cell experiments, conducted the bioinformatics and statistical analyses, prepared the figures, and drafted the manuscript. Y.H. and G.L. contributed to data organization, experimental validation, and manuscript revision. J.G., Q.Z., and Q.M. conceived and supervised the study. J.G., Q.Z., and Q.M. critically revised the manuscript.

## Funding

This work was supported by the Jinhua Science and Technology Research Program (2025‐4‐154) and the Jinhua Municipal Central Hospital Young and Middle‐aged Science and Technology Project (JY2024‐6‐11).

## Disclosure

All authors contributed to the article and approved the submitted version.

## Ethics Statement

Ethical review and approval were not required for this study because all publicly available transcriptomic datasets were obtained from the Gene Expression Omnibus (GEO) database, and the in vitro experiments were performed using established human cell lines. No human participants or animals were directly involved in this study.

## Conflicts of Interest

The authors declare no conflicts of interest.

## Supporting Information

Additional supporting information can be found online in the Supporting Information section.

## Supporting information


**Supporting Information 1** Figure S1: Gastric cancer tissues show coordinated remodeling of cellular composition and lineage‐specific functional programs. (A) Heatmap showing sample‐level cell type composition across normal and tumor tissues. (B) Paired comparison of patient‐level cell type proportions between normal and tumor tissues for major lineages. (C) Heatmap showing Hallmark pathway enrichment patterns across major cell types. (D–G) Enrichment summary plots showing representative GO biological process and Hallmark pathways in epithelial cells, mural cells, CD4+ T cells, and myeloid cells, respectively. (H) UMAP visualization of representative signature scores, including EMT, hypoxia, cytotoxicity, T‐cell exhaustion, CAF activation, and angiogenesis. (I) Sample‐level distribution of representative biological signatures across major cell types in normal and tumor tissues. (J) Heatmap showing cell type–by‐tissue signature patterns across major cellular compartments. (K) Comparison of representative epithelial‐related signatures between normal and tumor epithelial cells. (L) UMAP overlays showing the spatial distribution of representative epithelial programs, including EMT, hypoxia, MYC targets, and TGF‐beta signaling. (M) Heatmap showing epithelial signature patterns across samples. (N) Overview of immune‐related signature scores across major immune cell populations in normal and tumor tissues. (O) Heatmap showing immune signature patterns across immune cell types. (P) Absolute cell numbers of major cell types in normal and tumor tissues. (Q) Heatmap summarizing tumor‐versus‐normal differences in representative biological programs across cell types.

**Supporting Information 2** Figure S2: Reference‐centered CNV‐like inference identifies malignant epithelial states in gastric cancer. (A) UMAP of epithelial cells colored by tissue origin. (B) UMAP showing the distribution of reference‐centered CNV‐like scores across epithelial cells. (C) Distribution of CNV‐like scores in normal and tumor epithelial cells, with dashed lines indicating thresholds derived from the normal epithelial reference. (D) Average chromosome‐level CNV‐like profiles across reference normal, tumor CNV‐like‐low, tumor candidate, and tumor high‐confidence epithelial cells. (E) Heatmap of chromosome‐level CNV‐like signals across epithelial cells ordered by inferred class and CNV‐like burden. (F) UMAP showing classification of epithelial cells into reference normal, tumor CNV‐like‐low, tumor candidate, and tumor high‐confidence groups. (G) UMAP showing epithelial subtype annotation after epithelial‐focused reanalysis. (H) Subtype‐level landscape summarizing tumor fraction and predicted malignant cell fraction across epithelial subtypes. (I) Tumor sample composition of epithelial CNV‐like classes. (J) Volcano plot showing differential expression between tumor cells and nontumor epithelial cells at the cell level. (K) Volcano plot showing differential expression between tumor cells and tumor CNV‐like‐low epithelial cells using tumor‐only paired pseudobulk analysis. (L) Hallmark pathways associated with tumor epithelial cells. (M) GO biological processes associated with tumor epithelial cells. (N) Comparison of tumor‐related program scores across reference normal, tumor CNV‐like‐low, and tumor cells. (O) UMAP overlays showing the spatial distribution of representative tumor‐associated programs. (P) Dot plot showing representative tumor‐associated markers across epithelial cell classes. (Q) Heatmap showing sample‐level expression patterns of robust tumor markers across epithelial groups.

**Supporting Information 3** Figure S3: Fibroblasts occupy a central communication position and show tumor‐associated functional reprogramming. (A) Cell composition before and after balanced downsampling for CellChat analysis. (B) Matrix showing the number of significant ligand–receptor interactions between major cell groups. (C) Matrix showing the aggregated communication strength between major cell groups. (D) Centrality analysis of intercellular communication across major microenvironmental cell groups. (E) Heatmap showing outgoing communication strength from each cell group toward tumor epithelial, nontumor epithelial, and normal epithelial states. (F) Fibroblast‐centered summary of communication strength with partner cell groups. (G) Selected active signaling pathways involving fibroblasts and epithelial states. (H) Representative epithelial‐to‐fibroblast ligand–receptor interactions. (I) Fibroblast‐to‐epithelial communication map showing significant ligand–receptor pairs across epithelial states. (J) Top ligand–receptor pairs linking fibroblasts and tumor epithelial cells. (K) Chord diagrams showing representative pathway‐level communication between fibroblasts and epithelial states. (L) Volcano plot showing differential expression between tumor‐associated and normal fibroblasts. (M) Enrichment summary of representative GO biological process and Hallmark pathways in tumor‐associated fibroblasts. (N) UMAP overlays showing representative fibroblast‐related functional programs, including CAF ECM remodeling, myCAF‐like contractile features, iCAF‐like inflammatory features, and quiescent fibroblast states.

**Supporting Information 4** Figure S4: Deep characterization of the IGF1+ CXCL12+ CAF state. (A) UMAP highlighting IGF1+ CXCL12+ CAFs within the fibroblast compartment. (B) Feature plots showing representative markers enriched in IGF1+ CXCL12+ CAFs, including IGF1, CXCL12, IGFBP4, SFRP2, DPT, CCDC80, C3, CFD, and FBLN1. (C) Volcano plot showing differential expression between IGF1+ CXCL12+ CAFs and the remaining fibroblasts. (D) Volcano plot showing tumor‐associated transcriptional changes within IGF1+ CXCL12+ CAFs. (E) Enrichment analysis showing resistance‐relevant pathways associated with IGF1+ CXCL12+ CAFs and tumor‐versus‐normal changes within this subtype. (F) Heatmap showing functional state profiles across fibroblast subtypes. (G) Comparison of representative resistance‐supportive signature scores across fibroblast subtypes. (H) UMAP overlays showing representative programs enriched in IGF1+ CXCL12+ CAFs, including resistance support index, IGF1 axis, CXCL12 retention, and drug barrier ECM. (I) Paired comparison of IGF1+ CXCL12+ CAF proportions across normal and tumor tissues. (J) Heatmap showing sample‐level functional heterogeneity of IGF1+ CXCL12+ CAFs. (K) Dot plot showing marker expression across fibroblast subtypes. (L) Heatmap showing subtype‐average expression of representative IGF1+ CXCL12+ CAF markers.

**Supporting Information 5** Figure S5: External CF chemotherapy bulk evaluation of IGF1+ CXCL12+ CAF‐related programs. (A) Overview of the GSE14209 cohort showing paired pretreatment and posttreatment samples from 22 gastric cancer patients receiving cisplatin and fluorouracil combination chemotherapy. (B) Paired comparison of representative CAF‐related signature scores between posttreatment and pretreatment samples, including C3+ complement CAF, combined CAF resistance score, drug resistance, ECM barrier, IGF1+ CXCL12+ CAF core, and IGF1+ CXCL12+ CAF extended signatures. (C) Gene set enrichment analysis comparing posttreatment and pretreatment samples. (D) Heatmap showing patient‐level changes in representative CAF‐related signatures, with red indicating higher scores in posttreatment samples and blue indicating lower scores. (E) GSEA running score plot for the IGF1+ CXCL12+ CAF core signature in posttreatment versus pretreatment samples. (F) Summary of median changes in representative CAF‐related signature scores between posttreatment and pretreatment samples. (G) Correlation between IGF1 and CXCL12 expression in pretreatment and posttreatment samples.

**Supporting Information 6** Figure S6: ARPC1A knockdown suppresses migration and invasion of gastric cancer cells. (A) Representative images of wound‐healing assays in AGS and HGC‐27 cells transduced with shNC or three independent ARPC1A‐targeting shRNAs (shARPC1A‐1, shARPC1A‐2, and shARPC1A‐3) at 0 and 24 h. (B, C) Quantification of migration rates in AGS and HGC‐27 cells. (D) Representative images of Matrigel‐coated Transwell invasion assays in AGS and HGC‐27 cells under the indicated conditions. (E, F) Quantification of invasive capacity in AGS and HGC‐27 cells. Data are presented as mean ± SD from three independent experiments.  ^∗∗^ *p* < 0.01 and  ^∗∗∗^ *p* < 0.001 versus shNC.

**Supporting Information 7** Figure S7: ARPC1A knockdown reduces expression of drug‐resistance, antiapoptotic, and EMT‐related genes in gastric cancer cells. (A, B) qRT‐PCR analysis of ABCB1, ABCC1, BCL2, and MCL1 expression in AGS and HGC‐27 cells transduced with shNC or three independent ARPC1A‐targeting shRNAs (shARPC1A‐1, shARPC1A‐2, and shARPC1A‐3). (C, D) qRT‐PCR analysis of the epithelial marker CDH1 and the mesenchymal marker VIM in AGS and HGC‐27 cells under the indicated conditions. Relative mRNA expression levels were normalized to GAPDH and calculated using the 2^−*ΔΔ*Ct^ method. Data are presented as mean ± SD from three independent experiments.  ^∗^ *p* < 0.05,  ^∗∗^ *p* < 0.01, and  ^∗∗∗^ *p* < 0.001 versus shNC.

## Data Availability

The single‐cell RNA‐sequencing dataset analyzed in this study is publicly available in the Gene Expression Omnibus under Accession Number GSE206785. The external paired bulk transcriptome dataset used for treatment‐associated evaluation is publicly available in GEO under Accession Number GSE14209. TCGA‐STAD Pan‐Cancer Atlas mutation, copy‐number, expression, subtype, and clinical genomic data were accessed through cBioPortal. DepMap Public 24Q2 CRISPRGeneEffect and Model metadata were used for the virtual knockout analysis. Analysis scripts used for the single‐cell, bulk validation, CAF‐epithelial coupling, TCGA‐STAD genomic, and DepMap CRISPR analyses are available from the corresponding authors upon reasonable request and should be deposited in a public repository before publication. Experimental source data underlying the qRT‐PCR, cisplatin sensitivity, colony formation, apoptosis, wound‐healing, and Transwell assays are included in the Supporting Information or are available from the corresponding authors upon reasonable request.
