# Supplementary material for: Mutation‐Immune Crosstalk Contextualizes CAF‐Associated ARPC1A Programs and Cisplatin Response in Gastric Cancer
Source: Hum Mutat. 2026 Aug 19;2026:9607360. doi: 10.1155/humu/9607360 (PMC13490708; doi:10.1155/humu/9607360)
Supplement: Supplementary file 1 [file HUMU-2026-9607360-s001.pdf]

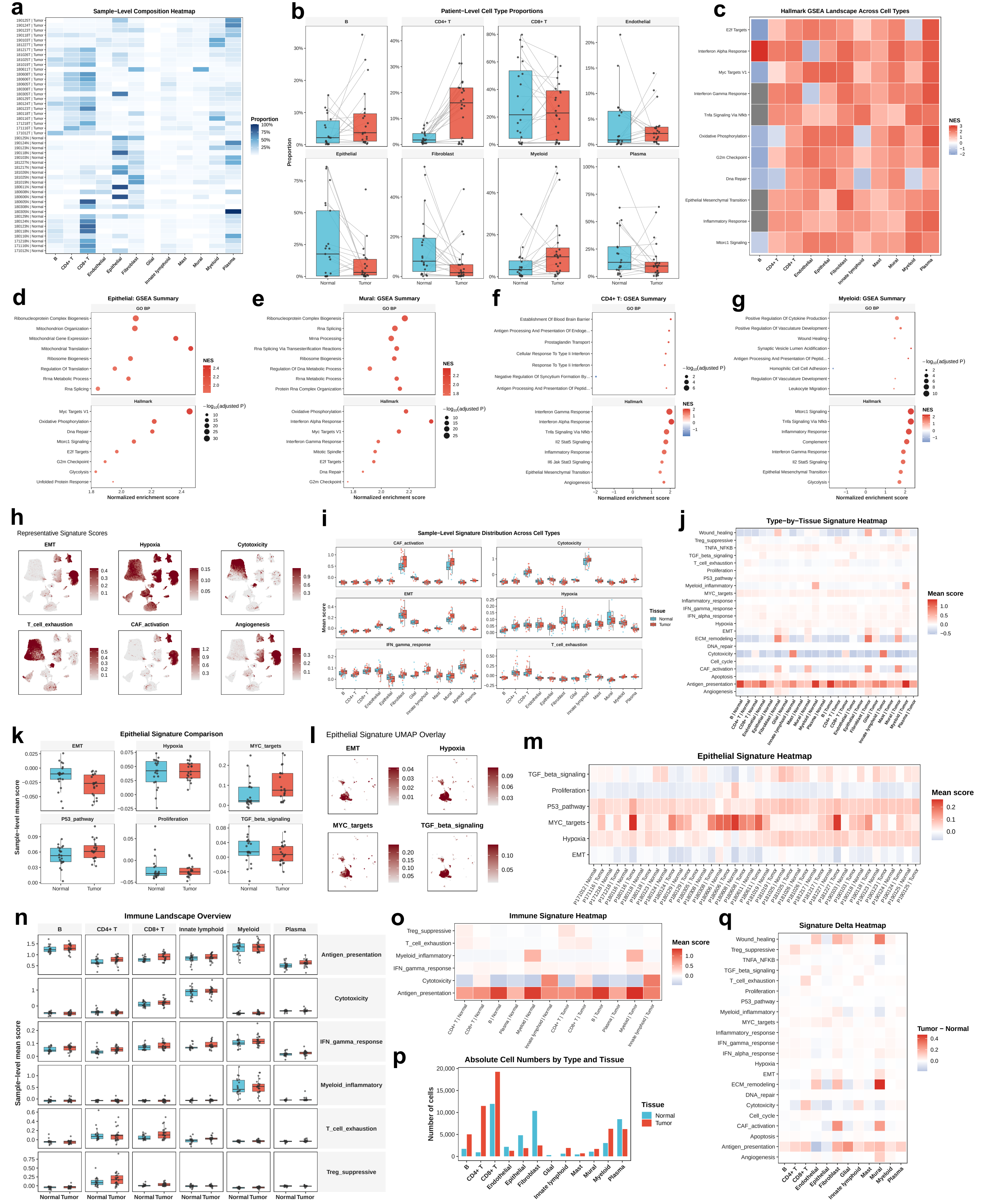
a
Sample-Level Composition Heatmap
Proportion
100%
75%
50%
25%
B
CD4+ T
CD8+ T
Endothelial
Epithelial
Fibroblast
Glial
Innate lymphoid
Mast
Mural
Myeloid
Plasma
b
Patient-Level Cell Type Proportions
Proportion
B
CD4+ T
CD8+ T
Endothelial
Epithelial
Fibroblast
Myeloid
Plasma
Normal
Tumor
c
Hallmark GSEA Landscape Across Cell Types
E2f Targets
Interferon Alpha Response
Myc Targets V1
Interferon Gamma Response
Tnfa Signaling Via Nfkb
Oxidative Phosphorylation
G2m Checkpoint
Dna Repair
Epithelial Mesenchymal Transition
Inflammatory Response
Mtorc1 Signaling
NES
d
Epithelial: GSEA Summary
GO BP
Ribonucleoprotein Complex Biogenesis
Mitochondrion Organization
Mitochondrial Gene Expression
Mitochondrial Translation
Ribosome Biogenesis
Regulation Of Translation
Rrna Metabolic Process
Rna Splicing
Hallmark
Myc Targets V1
Oxidative Phosphorylation
Dna Repair
Mtorc1 Signaling
E2f Targets
G2m Checkpoint
Glycolysis
Unfolded Protein Response
Normalized enrichment score
e
Mural: GSEA Summary
Ribonucleoprotein Complex Biogenesis
Rna Splicing
Mrna Processing
Rna Splicing Via Transesterification Reactions
Ribosome Biogenesis
Regulation Of Dna Metabolic Process
Rrna Metabolic Process
Protein Rna Complex Organization
Oxidative Phosphorylation
Interferon Alpha Response
Myc Targets V1
Interferon Gamma Response
Mitotic Spindle
E2f Targets
Dna Repair
G2m Checkpoint
f
CD4+ T: GSEA Summary
Establishment Of Blood Brain Barrier
Antigen Processing And Presentation Of Endoge...
Prostaglandin Transport
Cellular Response To Type Ii Interferon
Response To Type Ii Interferon
Negative Regulation Of Syncytium Formation By...
Antigen Processing And Presentation Of Peptid...
Interferon Gamma Response
Interferon Alpha Response
Tnfa Signaling Via Nfkb
Il2 Stat5 Signaling
Inflammatory Response
Il6 Jak Stat3 Signaling
Epithelial Mesenchymal Transition
Angiogenesis
g
Myeloid: GSEA Summary
Positive Regulation Of Cytokine Production
Positive Regulation Of Vasculature Development
Wound Healing
Synaptic Vesicle Lumen Acidification
Antigen Processing And Presentation Of Peptid...
Homophilic Cell Cell Adhesion
Regulation Of Vasculature Development
Leukocyte Migration
Mtorc1 Signaling
Tnfa Signaling Via Nfkb
Inflammatory Response
Complement
Interferon Gamma Response
Il2 Stat5 Signaling
Epithelial Mesenchymal Transition
Glycolysis
h
Representative Signature Scores
EMT
Hypoxia
Cytotoxicity
T_cell_exhaustion
CAF_activation
Angiogenesis
i
Sample-Level Signature Distribution Across Cell Types
CAF_activation
Cytotoxicity
EMT
Hypoxia
IFN_gamma_response
T_cell_exhaustion
Mean score
Tissue
Normal
Tumor
j
Type-by-Tissue Signature Heatmap
Wound_healing
Treg_suppressive
TNFA_NFKB
TGF_beta_signaling
T_cell_exhaustion
Proliferation
P53_pathway
Myeloid_inflammatory
MYC_targets
Inflammatory_response
IFN_gamma_response
IFN_alpha_response
Hypoxia
EMT
ECM_remodeling
DNA_repair
Cytotoxicity
Cell_cycle
CAF_activation
Apoptosis
Antigen_presentation
Angiogenesis
Mean score
k
Epithelial Signature Comparison
EMT
Hypoxia
MYC_targets
P53_pathway
Proliferation
TGF_beta_signaling
Sample-level mean score
l
Epithelial Signature UMAP Overlay
EMT
Hypoxia
MYC_targets
TGF_beta_signaling
m
Epithelial Signature Heatmap
TGF_beta_signaling
Proliferation
P53_pathway
MYC_targets
Hypoxia
EMT
Mean score
n
Immune Landscape Overview
B
CD4+ T
CD8+ T
Innate lymphoid
Myeloid
Plasma
Antigen_presentation
Cytotoxicity
IFN_gamma_response
Myeloid_inflammatory
T_cell_exhaustion
Treg_suppressive
Sample-level mean score
Normal Tumor
o
Immune Signature Heatmap
Treg_suppressive
T_cell_exhaustion
Myeloid_inflammatory
IFN_gamma_response
Cytotoxicity
Antigen_presentation
Mean score
p
Absolute Cell Numbers by Type and Tissue
Number of cells
Tissue
Normal
Tumor
q
Signature Delta Heatmap
Tumor − Normal
Wound_healing
Treg_suppressive
TNFA_NFKB
TGF_beta_signaling
T_cell_exhaustion
Proliferation
P53_pathway
Myeloid_inflammatory
MYC_targets
Inflammatory_response
IFN_gamma_response
IFN_alpha_response
Hypoxia
EMT
ECM_remodeling
DNA_repair
Cytotoxicity
Cell_cycle
CAF_activation
Apoptosis
Antigen_presentation
Angiogenesis
