# Supplementary material for: Mutation‐Immune Crosstalk Contextualizes CAF‐Associated ARPC1A Programs and Cisplatin Response in Gastric Cancer
Source: Hum Mutat. 2026 Aug 19;2026:9607360. doi: 10.1155/humu/9607360 (PMC13490708; doi:10.1155/humu/9607360)
Supplement: Supplementary file 2 [file HUMU-2026-9607360-s002.pdf]

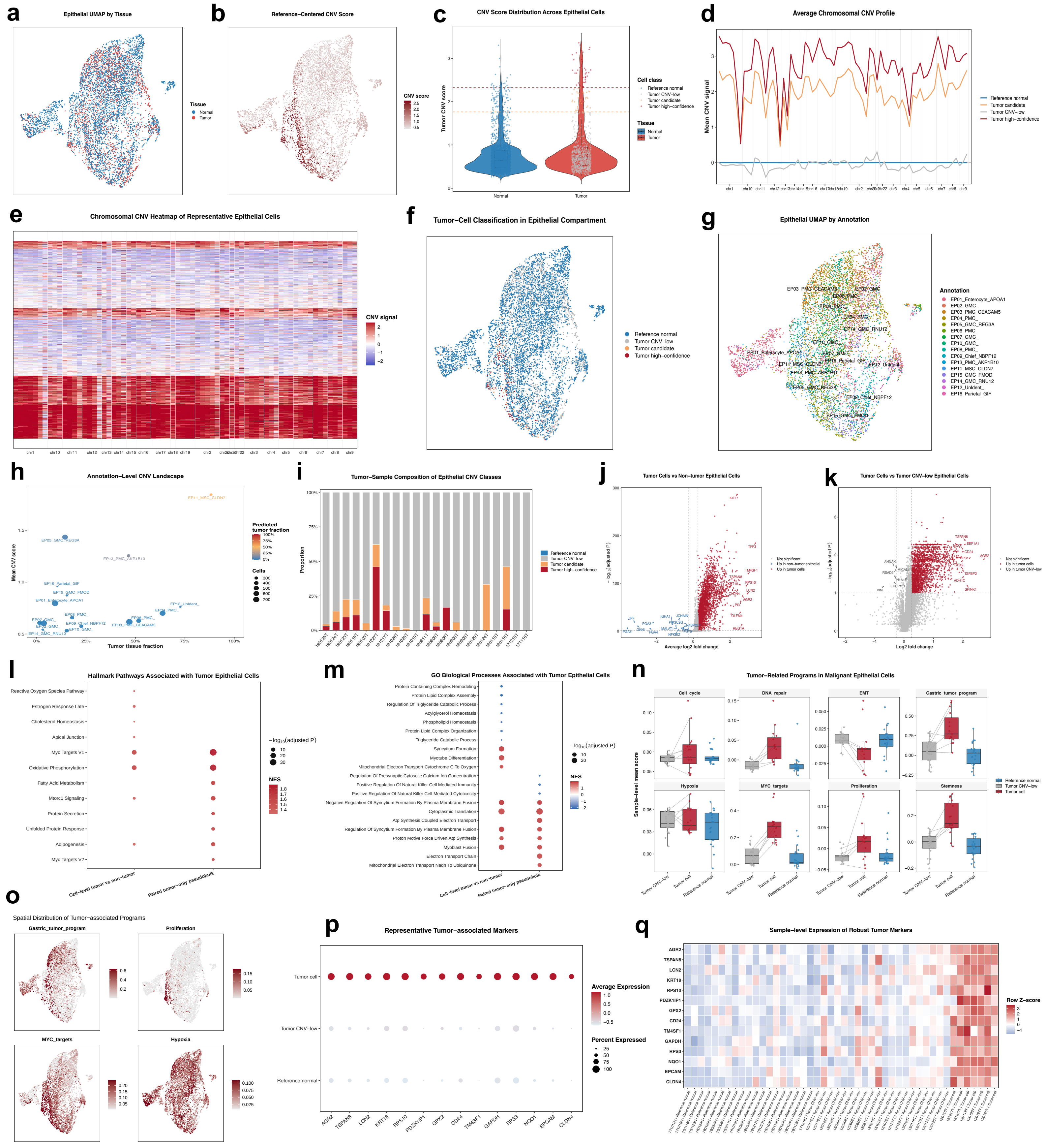
a
Epithelial UMAP by Tissue
b
Reference–Centered CNV Score
c
CNV Score Distribution Across Epithelial Cells
d
Average Chromosomal CNV Profile
e
Chromosomal CNV Heatmap of Representative Epithelial Cells
f
Tumor–Cell Classification in Epithelial Compartment
g
Epithelial UMAP by Annotation
h
Annotation–Level CNV Landscape
i
Tumor–Sample Composition of Epithelial CNV Classes
j
Tumor Cells vs Non–tumor Epithelial Cells
k
Tumor Cells vs Tumor CNV–low Epithelial Cells
l
Hallmark Pathways Associated with Tumor Epithelial Cells
m
GO Biological Processes Associated with Tumor Epithelial Cells
n
Tumor–Related Programs in Malignant Epithelial Cells
o
Spatial Distribution of Tumor–associated Programs
p
Representative Tumor–associated Markers
q
Sample–level Expression of Robust Tumor Markers
