# Supplementary material for: Mutation‐Immune Crosstalk Contextualizes CAF‐Associated ARPC1A Programs and Cisplatin Response in Gastric Cancer
Source: Hum Mutat. 2026 Aug 19;2026:9607360. doi: 10.1155/humu/9607360 (PMC13490708; doi:10.1155/humu/9607360)
Supplement: Supplementary file 3 [file HUMU-2026-9607360-s003.pdf]

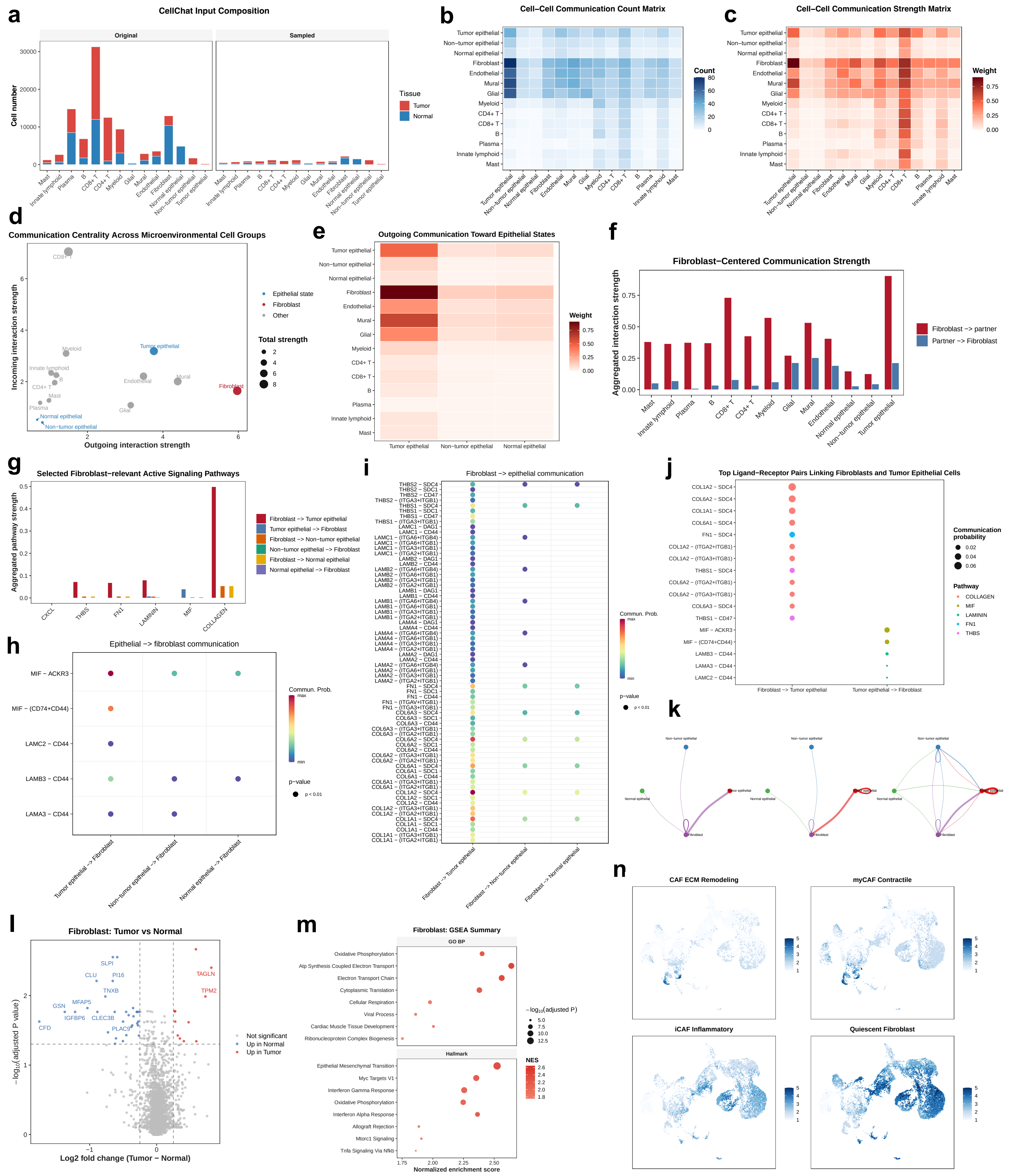
a
CellChat Input Composition
Original
Sampled
Cell number
Tissue
Tumor
Normal
Mast
Innate lymphoid
Plasma
B
CD8+ T
CD4+ T
Myeloid
Glial
Mural
Endothelial
Fibroblast
Normal epithelial
Non−tumor epithelial
Tumor epithelial
b
Cell−Cell Communication Count Matrix
Count
c
Cell−Cell Communication Strength Matrix
Weight
d
Communication Centrality Across Microenvironmental Cell Groups
Incoming interaction strength
Outgoing interaction strength
Epithelial state
Fibroblast
Other
Total strength
e
Outgoing Communication Toward Epithelial States
Weight
f
Fibroblast−Centered Communication Strength
Aggregated interaction strength
Fibroblast −> partner
Partner −> Fibroblast
g
Selected Fibroblast−relevant Active Signaling Pathways
Aggregated pathway strength
Fibroblast −> Tumor epithelial
Tumor epithelial −> Fibroblast
Fibroblast −> Non−tumor epithelial
Non−tumor epithelial −> Fibroblast
Fibroblast −> Normal epithelial
Normal epithelial −> Fibroblast
CXCL
THBS
FN1
LAMININ
MIF
COLLAGEN
h
Epithelial −> fibroblast communication
MIF − ACKR3
MIF − (CD74+CD44)
LAMC2 − CD44
LAMB3 − CD44
LAMA3 − CD44
Commun. Prob.
p-value
p < 0.01
Tumor epithelial −> Fibroblast
Non−tumor epithelial −> Fibroblast
Normal epithelial −> Fibroblast
i
Fibroblast −> epithelial communication
Fibroblast −> Tumor epithelial
Fibroblast −> Non−tumor epithelial
Fibroblast −> Normal epithelial
j
Top Ligand−Receptor Pairs Linking Fibroblasts and Tumor Epithelial Cells
COL1A2 − SDC4
COL6A2 − SDC4
COL1A1 − SDC4
COL6A1 − SDC4
FN1 − SDC4
COL1A2 − (ITGA2+ITGB1)
COL1A2 − (ITGA3+ITGB1)
THBS1 − SDC4
COL6A2 − (ITGA2+ITGB1)
COL6A2 − (ITGA3+ITGB1)
COL6A3 − SDC4
THBS1 − CD47
MIF − ACKR3
MIF − (CD74+CD44)
LAMB3 − CD44
LAMA3 − CD44
LAMC2 − CD44
Communication probability
Pathway
COLLAGEN
MIF
LAMININ
FN1
THBS
Fibroblast −> Tumor epithelial
Tumor epithelial −> Fibroblast
k
l
Fibroblast: Tumor vs Normal
SLPI
CLU
PI16
TNXB
MFAP5
GSN
IGFBP6
CLEC3B
CFD
PLAC9
TAGLN
TPM2
Not significant
Up in Normal
Up in Tumor
Log2 fold change (Tumor − Normal)
m
Fibroblast: GSEA Summary
GO BP
Oxidative Phosphorylation
Atp Synthesis Coupled Electron Transport
Electron Transport Chain
Cytoplasmic Translation
Cellular Respiration
Viral Process
Cardiac Muscle Tissue Development
Ribonucleoprotein Complex Biogenesis
Hallmark
Epithelial Mesenchymal Transition
Myc Targets V1
Interferon Gamma Response
Oxidative Phosphorylation
Interferon Alpha Response
Allograft Rejection
Mtorc1 Signaling
Tnfa Signaling Via Nfkb
NES
Normalized enrichment score
n
CAF ECM Remodeling
myCAF Contractile
iCAF Inflammatory
Quiescent Fibroblast
