# Supplementary material for: Mutation‐Immune Crosstalk Contextualizes CAF‐Associated ARPC1A Programs and Cisplatin Response in Gastric Cancer
Source: Hum Mutat. 2026 Aug 19;2026:9607360. doi: 10.1155/humu/9607360 (PMC13490708; doi:10.1155/humu/9607360)
Supplement: Supplementary file 4 [file HUMU-2026-9607360-s004.pdf]

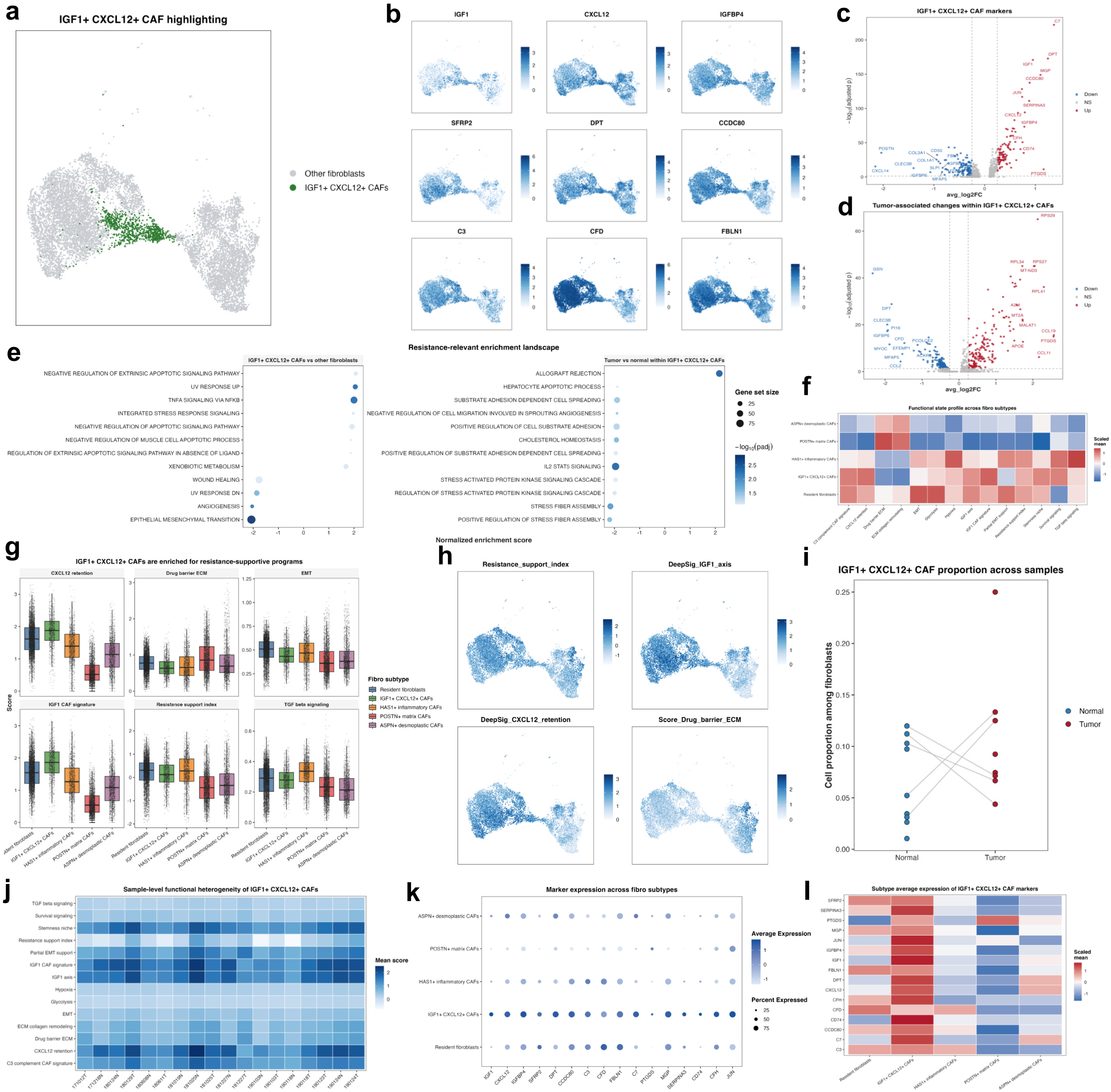
a
IGF1+ CXCL12+ CAF highlighting
Other fibroblasts
IGF1+ CXCL12+ CAFs
b
IGF1
CXCL12
IGFBP4
SFRP2
DPT
CCDC80
C3
CFD
FBLN1
c
IGF1+ CXCL12+ CAF markers
avg_log2FC
Down
NS
Up
d
Tumor-associated changes within IGF1+ CXCL12+ CAFs
e
Resistance-relevant enrichment landscape
IGF1+ CXCL12+ CAFs vs other fibroblasts
NEGATIVE REGULATION OF EXTRINSIC APOPTOTIC SIGNALING PATHWAY
UV RESPONSE UP
TNFA SIGNALING VIA NFKB
INTEGRATED STRESS RESPONSE SIGNALING
NEGATIVE REGULATION OF APOPTOTIC SIGNALING PATHWAY
NEGATIVE REGULATION OF MUSCLE CELL APOPTOTIC PROCESS
REGULATION OF EXTRINSIC APOPTOTIC SIGNALING PATHWAY IN ABSENCE OF LIGAND
XENOBIOTIC METABOLISM
WOUND HEALING
UV RESPONSE DN
ANGIOGENESIS
EPITHELIAL MESENCHYMAL TRANSITION
Tumor vs normal within IGF1+ CXCL12+ CAFs
ALLOGRAFT REJECTION
HEPATOCYTE APOPTOTIC PROCESS
SUBSTRATE ADHESION DEPENDENT CELL SPREADING
NEGATIVE REGULATION OF CELL MIGRATION INVOLVED IN SPROUTING ANGIOGENESIS
POSITIVE REGULATION OF CELL SUBSTRATE ADHESION
CHOLESTEROL HOMEOSTASIS
POSITIVE REGULATION OF SUBSTRATE ADHESION DEPENDENT CELL SPREADING
IL2 STAT5 SIGNALING
STRESS ACTIVATED PROTEIN KINASE SIGNALING CASCADE
REGULATION OF STRESS ACTIVATED PROTEIN KINASE SIGNALING CASCADE
STRESS FIBER ASSEMBLY
POSITIVE REGULATION OF STRESS FIBER ASSEMBLY
Normalized enrichment score
Gene set size
−log10(padj)
f
Functional state profile across fibro subtypes
g
IGF1+ CXCL12+ CAFs are enriched for resistance-supportive programs
CXCL12 retention
Drug barrier ECM
EMT
IGF1 CAF signature
Resistance support index
TGF beta signaling
Score
Fibro subtype
Resident fibroblasts
IGF1+ CXCL12+ CAFs
HAS1+ inflammatory CAFs
POSTN+ matrix CAFs
ASPN+ desmoplastic CAFs
h
Resistance_support_index
DeepSig_IGF1_axis
DeepSig_CXCL12_retention
Score_Drug_barrier_ECM
i
IGF1+ CXCL12+ CAF proportion across samples
Cell proportion among fibroblasts
Normal
Tumor
j
Sample-level functional heterogeneity of IGF1+ CXCL12+ CAFs
Mean score
k
Marker expression across fibro subtypes
Average Expression
Percent Expressed
l
Subtype average expression of IGF1+ CXCL12+ CAF markers
Scaled mean
