# Supplementary material for: Mutation‐Immune Crosstalk Contextualizes CAF‐Associated ARPC1A Programs and Cisplatin Response in Gastric Cancer
Source: Hum Mutat. 2026 Aug 19;2026:9607360. doi: 10.1155/humu/9607360 (PMC13490708; doi:10.1155/humu/9607360)
Supplement: Supplementary file 5 [file HUMU-2026-9607360-s005.pdf]

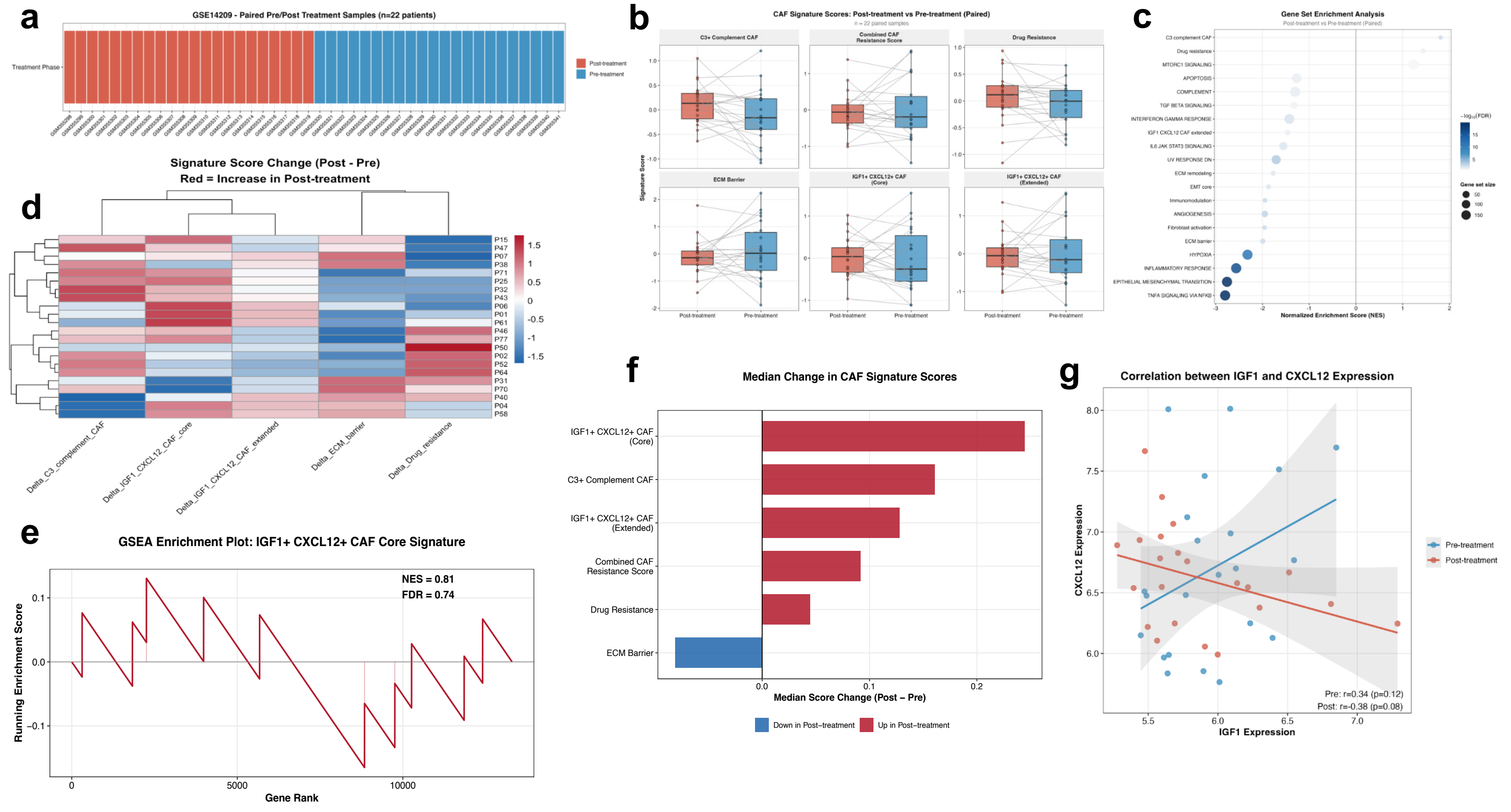

a
GSE14209 - Paired Pre/Post Treatment Samples (n=22 patients)
Treatment Phase
Post-treatment
Pre-treatment
b
CAF Signature Scores: Post-treatment vs Pre-treatment (Paired)
n = 22 paired samples
C3+ Complement CAF
Combined CAF Resistance Score
Drug Resistance
ECM Barrier
IGF1+ CXCL12+ CAF (Core)
IGF1+ CXCL12+ CAF (Extended)
Signature Score
Post-treatment
Pre-treatment
c
Gene Set Enrichment Analysis
Post-treatment vs Pre-treatment (Paired)
C3 complement CAF
Drug resistance
MTORC1 SIGNALING
APOPTOSIS
COMPLEMENT
TGF BETA SIGNALING
INTERFERON GAMMA RESPONSE
IGF1 CXCL12 CAF extended
IL6 JAK STAT3 SIGNALING
UV RESPONSE DN
ECM remodeling
EMT core
Immunomodulation
ANGIOGENESIS
Fibroblast activation
ECM barrier
HYPOXIA
INFLAMMATORY RESPONSE
EPITHELIAL MESENCHYMAL TRANSITION
TNFA SIGNALING VIA NFKB
Normalized Enrichment Score (NES)
-log10(FDR)
Gene set size
d
Signature Score Change (Post - Pre)
Red = Increase in Post-treatment
P15
P47
P07
P38
P71
P25
P32
P43
P06
P01
P61
P46
P77
P50
P02
P52
P64
P31
P70
P40
P04
P58
Delta_C3_complement_CAF
Delta_IGF1_CXCL12_CAF_core
Delta_IGF1_CXCL12_CAF_extended
Delta_ECM_barrier
Delta_Drug_resistance
e
GSEA Enrichment Plot: IGF1+ CXCL12+ CAF Core Signature
NES = 0.81
FDR = 0.74
Running Enrichment Score
Gene Rank
f
Median Change in CAF Signature Scores
IGF1+ CXCL12+ CAF (Core)
C3+ Complement CAF
IGF1+ CXCL12+ CAF (Extended)
Combined CAF Resistance Score
Drug Resistance
ECM Barrier
Median Score Change (Post – Pre)
Down in Post–treatment
Up in Post–treatment
g
Correlation between IGF1 and CXCL12 Expression
CXCL12 Expression
IGF1 Expression
Pre: r=0.34 (p=0.12)
Post: r=-0.38 (p=0.08)
Pre-treatment
Post-treatment
