# Supplementary material for: Mutation‐Immune Crosstalk Contextualizes CAF‐Associated ARPC1A Programs and Cisplatin Response in Gastric Cancer
Source: Hum Mutat. 2026 Aug 19;2026:9607360. doi: 10.1155/humu/9607360 (PMC13490708; doi:10.1155/humu/9607360)
Supplement: Supplementary file 6 [file HUMU-2026-9607360-s006.pdf]

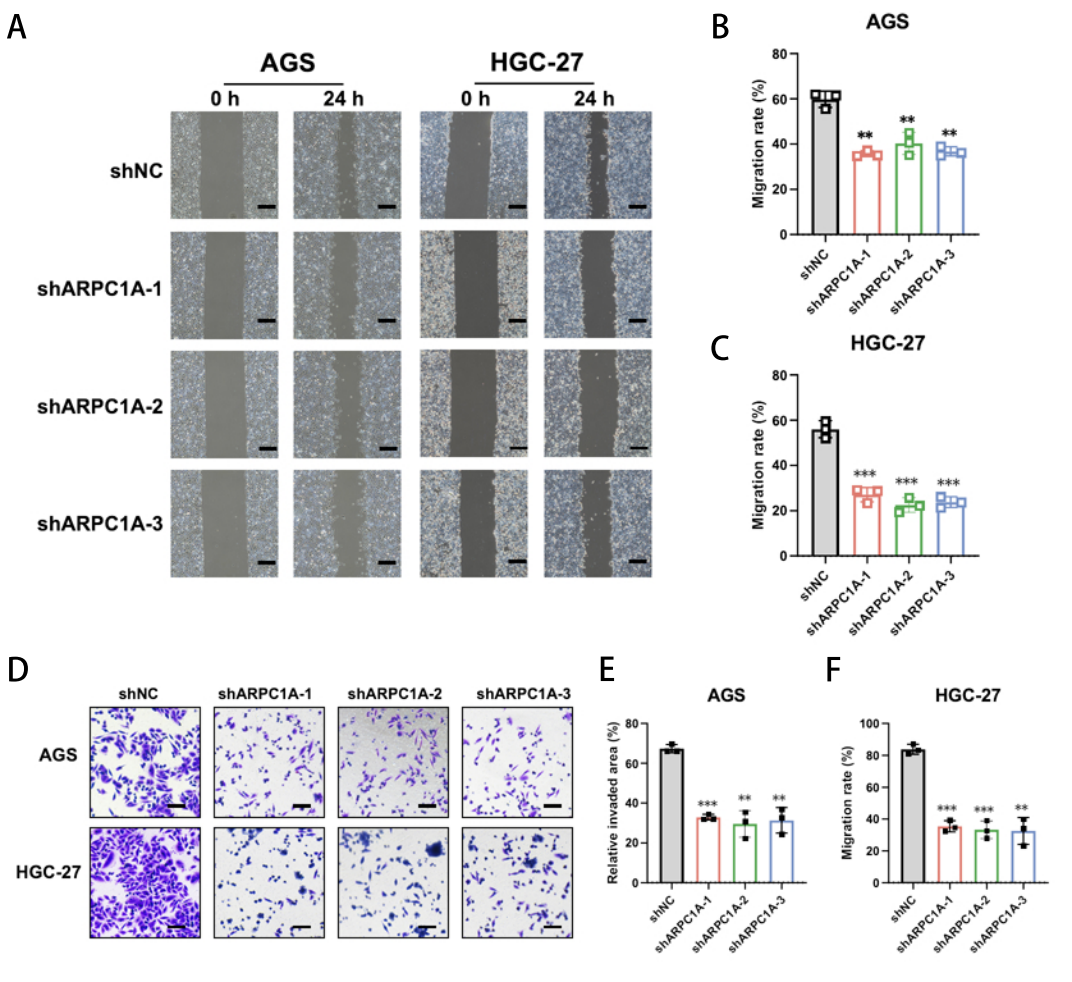

A
AGS
HGC-27
0 h
24 h
0 h
24 h
shNC
shARPC1A-1
shARPC1A-2
shARPC1A-3
B
AGS
Migration rate (%)
0
20
40
60
80
**
**
**
shNC
shARPC1A-1
shARPC1A-2
shARPC1A-3
C
HGC-27
Migration rate (%)
0
20
40
60
80
***
***
***
shNC
shARPC1A-1
shARPC1A-2
shARPC1A-3
D
shNC
shARPC1A-1
shARPC1A-2
shARPC1A-3
AGS
HGC-27
E
AGS
Relative invaded area (%)
0
20
40
60
80
***
**
**
shNC
shARPC1A-1
shARPC1A-2
shARPC1A-3
F
HGC-27
Migration rate (%)
0
20
40
60
80
100
***
***
**
shNC
shARPC1A-1
shARPC1A-2
shARPC1A-3
