# Supplementary material for: Mutation‐Immune Crosstalk Contextualizes CAF‐Associated ARPC1A Programs and Cisplatin Response in Gastric Cancer
Source: Hum Mutat. 2026 Aug 19;2026:9607360. doi: 10.1155/humu/9607360 (PMC13490708; doi:10.1155/humu/9607360)
Supplement: Supplementary file 7 [file HUMU-2026-9607360-s007.pdf]

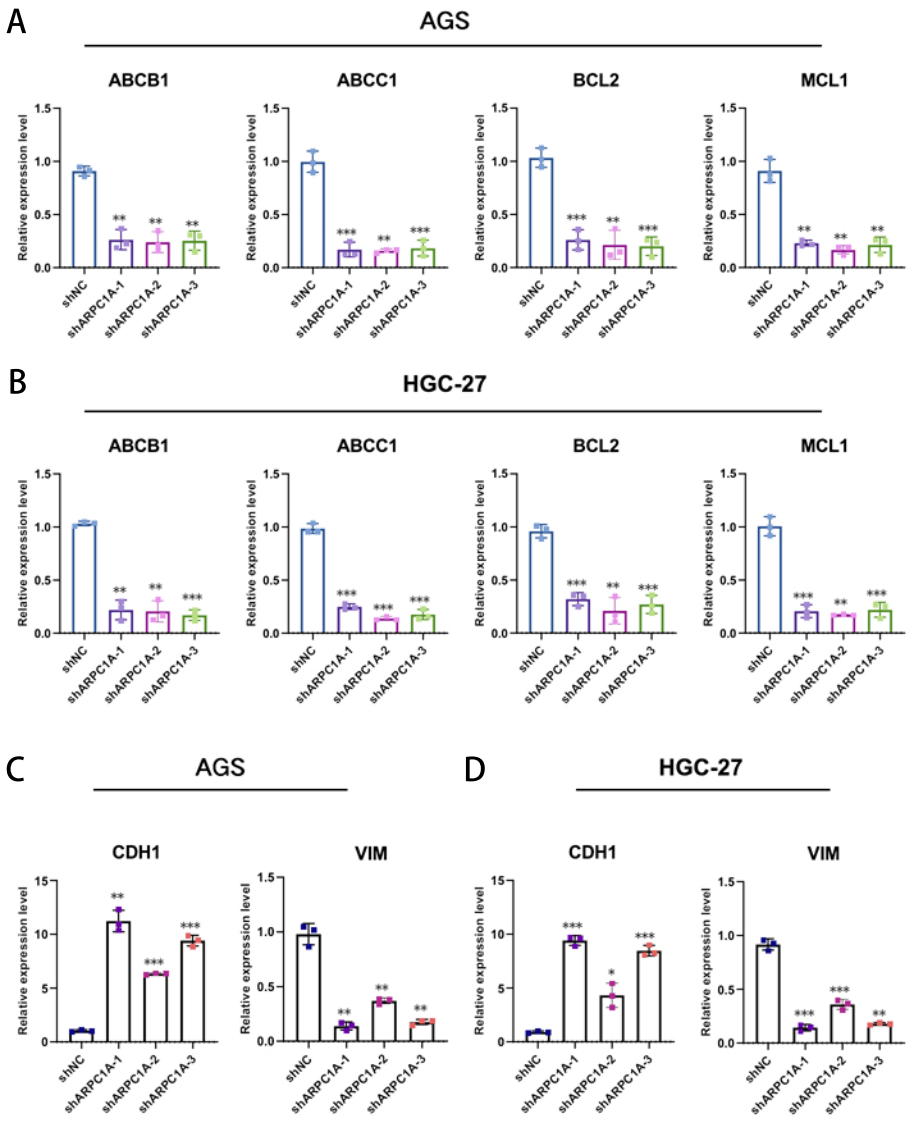
A
AGS
ABCB1
ABCC1
BCL2
MCL1
B
HGC-27
ABCB1
ABCC1
BCL2
MCL1
C
AGS
CDH1
VIM
D
HGC-27
CDH1
VIM
Relative expression level
shNC
shARPC1A-1
shARPC1A-2
shARPC1A-3
